# Heterogeneity of genetic architecture of body size traits in a free‐living population

**DOI:** 10.1111/mec.13146

**Published:** 2015-03-30

**Authors:** Camillo Bérénos, Philip A. Ellis, Jill G. Pilkington, S. Hong Lee, Jake Gratten, Josephine M. Pemberton

**Affiliations:** ^1^ Institute of Evolutionary BiologyEdinburghEH9 3FLUK; ^2^ Queensland Brain InstituteThe University of QueenslandBrisbaneQld4072Australia

**Keywords:** admixture, body size, genetic architecture, genome partitioning, genome‐wide association, genomics

## Abstract

Knowledge of the underlying genetic architecture of quantitative traits could aid in understanding how they evolve. In wild populations, it is still largely unknown whether complex traits are polygenic or influenced by few loci with major effect, due to often small sample sizes and low resolution of marker panels. Here, we examine the genetic architecture of five adult body size traits in a free‐living population of Soay sheep on St Kilda using 37 037 polymorphic SNPs. Two traits (jaw and weight) show classical signs of a polygenic trait: the proportion of variance explained by a chromosome was proportional to its length, multiple chromosomes and genomic regions explained significant amounts of phenotypic variance, but no SNPs were associated with trait variance when using GWAS. In comparison, genetic variance for leg length traits (foreleg, hindleg and metacarpal) was disproportionately explained by two SNPs on chromosomes 16 (s23172.1) and 19 (s74894.1), which each explained >10% of the additive genetic variance. After controlling for environmental differences, females heterozygous for s74894.1 produced more lambs and recruits during their lifetime than females homozygous for the common allele conferring long legs. We also demonstrate that alleles conferring shorter legs have likely entered the population through a historic admixture event with the Dunface sheep. In summary, we show that different proxies for body size can have very different genetic architecture and that dense SNP helps in understanding both the mode of selection and the evolutionary history at loci underlying quantitative traits in natural populations.

## Introduction

Phenotypic change in traits thought to be under directional selection is often absent in long‐term studies of wild populations and sometimes goes in the opposite direction to what is expected (Larsson *et al*. 1998; Kruuk *et al*. 2001; Ozgul *et al*. 2009). Explaining to what extent evolutionary or ecological processes are responsible for observed micro‐evolutionary change, or the lack thereof, has often proven very difficult (Morrissey *et al*. 2012). For example, proxies for body size such as height, tarsus length or body mass are associated with fitness measures in a wide range of taxa, so that body size is under positive selection in the majority of published studies (Kingsolver & Pfennig 2004; Kingsolver & Diamond 2011). Give that body size is heritable, a phenotypic response to selection, in terms of an increase in body size might be expected, but in fact is rarely observed. Stasis or even a decline in body size has been reported in several natural populations, often in the face of positive directional selection (Larsson *et al*. 1998; Merilä *et al*. 2001).

There are several major explanations for the discrepancy between expectation and observation. First, even relatively long‐term individual‐based studies of wild populations may be of insufficient duration to detect evolutionary responses. Typically, these studies are conducted in bird or mammal populations, which often have relatively long generation times, resulting in observations on an ecological rather than on an evolutionary timescale. Second, the observed covariance between body size and fitness may be predominantly due to environmental factors rather than an underlying causal genetic covariance (Kruuk *et al*. 2002). This would preclude evolutionary change, even if body size is phenotypically associated with fitness. While there is ample evidence for a phenotypic association between body size and fitness, no studies have shown convincing evidence for a genetic correlation between body size and fitness. For example, in the Soay sheep (*Ovis aries*) on St Kilda, a lack of genetic correlation between body size and fitness indicates that no net genetic response to selection can be expected (Morrissey *et al*. 2012).

Quantitative genetics provides a methodology to assess whether the relationship between body size and fitness is causal, typically using pedigrees to estimate the relatedness between individuals (Hadfield *et al*. 2010; Morrissey *et al*. 2012). A limitation of this methodology is that it can neither identify the number nor the physical positions of causal genomic regions contributing to trait variation, and nor can it reveal how they affect fitness. While body size is usually considered a classical polygenic trait and is probably influenced by many genes (Visscher *et al*. 2007), it is possible (i) that body size traits can be influenced by a few genes with large effect, many genes with small effect or the entire spectrum between these two extremes of the theoretically possible distribution and more importantly (ii) that the degree to which different proxies for body size are controlled by the same regions varies between pairs of traits despite being superficially correlated at a phenotypic level. Examining which traits underlie body size in specific or complex traits in general can also contribute to our understanding of what drives or limits the evolution of those traits. Different causal genomic regions can differ in the magnitude and sign of their effects on fitness, hence possibly explaining the widespread observation of stasis.

In the last decade, studies using genome‐wide association (GWAS), have discovered hundreds of variants underlying complex traits in human and livestock genetics (Goddard & Hayes 2009; Hindorff *et al*. 2009; Visscher *et al*. 2012). Despite this success, it has also emerged that the variants discovered generally have very small effects on trait values or disease risk, and even in large‐scale studies, the vast majority of the additive genetic variance for complex traits remains unexplained when using this approach (Manolio *et al*. 2009; Gibson 2010). In humans, using relatedness at many single nucleotide polymorphism (SNP) markers in a mixed‐model framework, up to half of the heritable variation for a range of complex traits is accounted for by common SNP markers (Yang *et al*. 2010). This is substantially more than the proportion of variance explained by genome‐wide significant SNPs alone (Lango Allen *et al*. 2010) and suggests that most of the genetic variance is due to the variants which are either rare or have small effects on phenotypes. Using chromosome‐specific SNP‐based relatedness matrices, it was shown that many chromosomes contributed to genetic variance in a suite of complex traits and that the phenotypic variance explained by a chromosome scaled with its physical length (Visscher *et al*. 2007; Yang *et al*. 2011b). Both findings support the polygenic models of quantitative traits as they are in agreement with the idea that many genes, each with a relatively small effect on trait variation, are scattered randomly throughout the genome and as a result, larger chromosomes generally harbour more causal genes than smaller chromosomes. A more recently developed method which can be applied to dense genomic data is regional heritability mapping which falls in between both chromosome partitioning and GWAS and has led to promising results in both human and livestock data sets (Nagamine *et al*. 2012; Riggio *et al*. 2013).

The above observations in humans and domestic animals are in contrast with results from wild animal populations, where the few published studies to date generally discovered major effect quantitative trait loci (QTL), with single regions often explaining >50% of the heritable variation for quantitative traits (Slate 2013). There are a number of possible explanations for this contrast. A meta‐analysis found that QTL effect sizes are inversely correlated with sample sizes, suggesting that QTL effect sizes in natural populations are universally inflated due to small sample sizes, a phenomenon termed the Beavis effect (Beavis 1994). Second, most wild animal studies have used linkage rather than association mapping (Slate 2013). Linkage mapping typically suffers from poor resolution – QTL confidence intervals can span large segments of the genome – and can thus harbour multiple causal loci (Slate *et al*. 2010). Third, many wild animal studies use relatively sparse marker density which also limits power. Modern genetic tools such as large‐scale SNP genotyping offer much greater resolution as a result of the increased marker density. Fortunately, they are increasingly available for wild populations (Johnston *et al*. 2011; Hagen *et al*. 2013; Robinson *et al*. 2013; Santure *et al*. 2013; Kawakami *et al*. 2014), and their use may lead to improvements in our understanding of the genetic architecture of body size and other morphological traits in natural populations (Slate *et al*. 2010).

The Soay sheep (*Ovis aries*) is a primitive sheep breed which has lived unmanaged on St Kilda for thousands of years. Body size is heritable (Milner *et al*. 2000; Wilson *et al*. 2007), and genetic correlations among five proxies for adult body size range between 0.27 and 0.94 (Table S1, Supporting information; Bérénos *et al*. 2014). Body size is positively associated with fitness components such as juvenile overwinter survival and fitness predictors such as parasite resistance (Coltman *et al*. 2001). Despite this, body size has declined since the start of the study period in 1985 (Wilson *et al*. 2007; Ozgul *et al*. 2009). Recent studies have shown that the association between size and fitness is nongenetic (Ozgul *et al*. 2009; Morrissey *et al*. 2012) and that the reduction in body size is probably due to increased overwinter survival of smaller individuals as a result of changing environmental conditions (Ozgul *et al*. 2009).

To date, most of our knowledge of the genetic architecture of Soay sheep body size has been obtained using pedigree‐derived estimates (Wilson *et al*. 2007) and more recently, genome‐wide SNP marker‐derived estimates of relatedness in a quantitative genetic framework (Bérénos *et al*. 2014). A more precise understanding of how genetic variation for the various body size traits is distributed throughout the genome is now needed. Previous analyses using linkage mapping revealed one significant QTL and several suggestive QTL underlying various body size traits (Beraldi *et al*. 2007), but it is expected that the current genomic tools will have less bias and far superior power and precision to unravel the genetic architecture of traits in Soay sheep.

The objectives of this study were to: (i) partition additive genetic variance in body size between chromosomes and compare the *V*_A_ explained by individual chromosomes with their length; (ii) partition *V*_A_ between genomic regions; (iii) identify which SNPs are associated with body size; (iv) test whether any identified SNPs are associated with fitness and if so the nature of selection involved; and (v) infer the origin of haplotypes surrounding any SNPs associated with body size. We expect that if the genetic correlation between traits is strong (i.e. among leg length traits), the same chromosomes, regions or SNPs contribute to phenotypic variance in those traits. Similarly, if the genetic correlation is weaker (e.g. between leg length traits, weight and jaw length), we expect that there will be less overlap in the chromosomes, regions or SNPs explaining significant amounts of trait variance.

## Methods

### Phenotype and genotype data

The Soay sheep is a primitive breed which lives in an unmanaged state in the St Kilda archipelago, NW Scotland (Clutton‐Brock & Pemberton 2004). Although they are probably to be direct descendants of the first sheep brought to the British Isles during the Bronze Age, in the 19th century, they also experienced an admixture event with the now extinct Dunface sheep breed (Feulner *et al*. 2013) and through this or other such events, they acquired markers of the second wave of sheep domestication (Chessa *et al*. 2009). Sheep resident in the Village Bay area, where approximately one‐third of the sheep inhabiting the island of Hirta are found, have been the subject of a long‐term individual‐based study since 1985. Following (Bérénos *et al*. 2014), we studied the genetic architecture of five proxies of body size, three of which are measured on live animals during the annual August expedition (foreleg, hindleg, weight) and two of which are taken on postmortem skeletal parts (metacarpal, jaw). More detailed information about trait measurements can be found in Beraldi *et al*. 2007;. Foreleg, hindleg, metacarpal and jaw are all length measures (mm), and weight is measured in kg. The identity of all measured sheep is known as all sheep are ear‐tagged when they are first captured, which is generally within a few days after birth. For adult measures, heritabilities range from 0.26 to 0.59; genetic correlations are modest between most of the traits (0.29–0.54), but are very high between the three leg length measures (0.89–0.94, all estimates obtained using genomic estimates of relatedness, Bérénos *et al*.^2014^, Table S1, Supporting information).

Genotype information at 37 037 informative autosomal SNP markers on the Ovine SNP50 BeadChip [Illumina, for more information about locus quality control and marker characteristics see (Bérénos *et al*. 2014)] was available for a total of 5805 sheep spanning the entire duration of the study period 1985–2012. SNP positions were obtained from v3.1 of the sheep genome: http://www.livestockgenomics.csiro.au.

### Genomic analysis of body size

In Soay sheep, the heritability of body size increases with age and the proportion of variance explained by maternal effects decreases with age (Wilson *et al*. 2007). In order to maximize power to detect associations between genomic regions and trait values, we restricted our genomic analyses to trait measures collected in adult sheep. For the live measures (foreleg, hindleg and weight), these were individuals captured aged 28 months and older, (corresponding to the adult age class in Bérénos *et al*. 2014) and for the skeletal measures (metacarpal and jaw), these were individuals found dead at 14 months or older (corresponding to the yearling and adult age class in Bérénos *et al*. 2014). As the vast majority of the mortality happens during late winter/early spring, <1% of sheep with skeletal data had experienced less than two full summers, ~25% of sheep had experienced two summers, with the remaining sheep having experienced three summers or more. All the analyses presented were robust to any differences in age composition between the August expedition and skeletal data.

#### Partitioning of genetic variance between chromosomes and genomic regions

Phenotypic variance for body size traits was partitioned into genetic and environmental variance components using univariate animal models, which can fit both fixed and random effects (Kruuk 2004). Sex and age at measurement were treated as multilevel factors and were included as fixed effects in all analyses.

We analysed trait variance using the following models:

(eqn 1) $$y=X\beta +Z_{1}a+Z_{r}u_{r}+e$$

(eqn 2) $$y=X\beta +Z_{1}c_{i}+Z_{2}{\mathrm{ra}}_{i}+Z_{r}u_{r}+e$$

(eqn 3) $$y=X\beta +Z_{2}{\mathrm{ra}}_{i}+Z_{r}u_{r}+e.$$

where **y** is the vector of phenotypic observations for all individuals, **X** is an incidence matrix linking individual records with vector of fixed effects β*;* **Z**_1_, **Z**_2_ and **Z**_*r*_ are incidence matrices which are used to relate random effects to the individual trait records. **a** is the vector of the additive genetic effects accounted for by genomic relatedness at all autosomal markers, **c** is the vector of the additive genetic effects explained by genomic region *i* and **ra** is the vector of the additive genetic effects explained by genomic relatedness at all autosomal markers except for those found in region *i*. Additional random effects **u**_r_ varied between traits and are fitted with their own corresponding incidence matrix **Z**_r_. Birth year was fitted as a random effect in all models. For adult August phenotypic data, year of measurement and permanent environment effects were also fitted as random effects in all models. Maternal effects were not fitted as they explain very little of the phenotypic variance in adult size traits (Bérénos *et al*. 2014), and as maternal identity was not known for all animals, fitting a maternal effect would have led to lower sample sizes. Our data sets comprised of approximately 2550 measures on 900 individuals for August catch traits and 940–1020 individuals for skeletal traits.

Using Model (1), we estimated the total genomic heritability. Using Model (2), we partitioned variance between genomic regions at two levels of increasing precision. We first partitioned variance between chromosomes, by fitting a genomic‐relatedness matrix (GRM) for chromosome *i* and a GRM for all remaining autosomal SNPs. Second, we partitioned phenotypic variance between regions of 150 adjacent SNP markers, again by fitting a GRM calculated using all SNPs in region *i* together with a GRM calculated using all remaining autosomal markers, using the same model structure as above (Model 2, similar to Nagamine *et al*. 2012). Regions were created using a sliding window approach, where regions of 150 adjacent SNP markers started 75 SNPs apart. For example, for each chromosome, region 1 consisted of SNPs 1–150, region 2 consisted of SNP 76–225 and so forth. At the end of each chromosome, this resulted in some regions having fewer than 150 SNPs; only regions containing more than 112 SNPs were used in the analyses (Table S2, Supporting information).

The genomic‐relatedness matrices (GRM) between all pairs of individuals included in the models were estimated in gcta v1.04 (Yang *et al*. 2010, 2011a) and weighted using allele frequency estimates calculated using genotype information for a total of 5805 sheep. Relatedness estimates were shrunk using the *–adj 0* command in gcta v1.04. Adjustments were needed to adjust for sampling error in estimating relatedness using a finite number of markers in genomic regions. No adjustments were made for potential differences in allelic spectrum between genotyped SNPs and causal variants. Variance components for models which converged without adjustments differed very little from models where adjustments were made, suggesting that adjustments for sampling error did not introduce bias. Significance of the proportion of phenotypic variance attributed to chromosomes or genomic regions was tested by comparing the log‐likelihood of model 2 with the log‐likelihood of model (3) using a log‐likelihood test (LRT) assuming a chi‐square distribution with one degree of freedom. When partitioning between 150‐SNP regions, not all models converged. Model convergence was obtained for 452 (foreleg), 455 (hindleg), 461 (metacarpal), 449 (weight) and 452 (jaw) of the 468 models attempted. A region was considered to have a significant effect on trait variation if, first, *P* was lower than α = 0.05 divided by half the number of genomic regions for which model convergence was reached. No adjustments for multiple testing were made when partitioning phenotypic variance between chromosomes. All models were run in the asreml‐r package for r (Gilmore *et al*. 2009).

#### Testing for associations between individual SNPs and trait values

Genome‐wide association mapping was performed to test for associations between SNPs and the five body size traits. We first analysed trait variation using a mixed model (Model 1) to account for both random effects (such as whole‐genome relatedness, the permanent environment and year of measurement) and fixed effects (sex, age at measurement). GRM were not adjusted for sampling errors or differences in allelic spectrum between SNPs and causal variants. We then tested for association between residuals extracted from these mixed models and individual SNPs using the *qtscore* function in the r package GenABEL (Aulchenko *et al*. 2007b). For traits measured during the August catch, we used mean residual values as repeated measurements were available for some sheep. Significance threshold was adjusted for multiple testing using a Bonferroni correction, with α = 0.05 divided by the number of SNPs, leading to a genome‐wide significance threshold of *P* = 1.35 × 10^−6^. This approach follows the GRAMMAR method and is known to have lower power than mixed‐model association methods where polygenic and SNP effects are estimated simultaneously (Aulchenko *et al*. 2007a). Mixed‐model association methods are generally much more computationally demanding than GRAMMAR, and although more efficient mixed‐model methods have been developed recently, they are not able to deal with repeated measures and nongenetic random effects (Yang *et al*. 2014). Performing GWAS using a fully specified mixed model in ASREML would be computationally unfeasible. Hence, for each region which contained SNPs near or exceeding genome‐wide significance, we estimated unbiased effect sizes of the most highly associated SNP in asreml‐r using an extended version of Model (1), where SNP genotype, expressed as the number of minor alleles carried by an individual, was fitted as an additional covariate. Phenotypic variance explained by significant SNPs was then estimated using the following equation:

$$V_{\mathrm{SNP}}=2pqa^{2}$$

where *p* and *q* are the frequencies of the major and minor allele, and *a* is additive SNP effect (Falconer & Mackay 1996)

The proportion of phenotypic and genetic variance explained by the focal SNP was calculated by the ratio of *V*_SNP_ to total phenotypic variance (*V*_P_) or additive genetic variance (*V*_A_), respectively.

### Testing for fitness differences at QTL loci

We first tested whether there were fitness differences between genotypes at genome‐wide significant SNP loci, by analysing two separate annual fitness components (annual survival: AS and annual number of recruits: AR) and two measures of lifetime fitness (lifetime breeding success: LBS and lifetime number of recruits: LR). We used a pedigree derived by SNP‐based parentage inference (Pedigree 2 in Bérénos *et al*. 2014) to calculate the number of lambs or recruits produced per sheep. LBS was defined as the number of lambs born to a female or the number of lambs sired by a male. To avoid bias in LBS estimates due to either sparse pedigree data or censoring of animals still alive, LBS was calculated for all individuals born between 1990 and 2003 using parentage data up until 2012. The data included only one male and a handful of females born in or before 2003 and still alive in 2012, all of which were far past their reproductive peak.

For each sheep year *j,* AS was defined as binary response to whether or not an individual survived past November 1st in year *j*, and AR was defined for each sheep year *j* as the number of offspring born in year *j* which were still alive on November 1st. LR was calculated as the total number of recruits (offspring lambs who survived past November 1st in their first year) produced during the lifetime of an individual. For each individual, AS, AR and LR were only estimated for years an individual was part of the study population, or alternatively for males, when a male was observed in the study area during the preceding rut. As we only study a part of the entire island population and individuals are able to roam and reproduce outside the boundaries of the study system, inevitably, our fitness estimates are downwardly biased. This downward bias is relatively modest for females, as they are philopatric, but may be larger for males which exhibit much lower natal fidelity, and who may be siring offspring outside the study area (Coltman *et al*. 2003). Because AS, AR and LR only taken into account fitness for years an individual was part of the study population, this downward bias is less than for LBS. Sample sizes per sex ranged between 802 and 1418. All fitness measures were analysed using generalized linear mixed models (GLMM) with a Bayesian approach using Markov chain Monte Carlo (MCMC) algorithms in the r package MCMCglmm (Hadfield 2010). For models of AS and AR, fixed effects included litter size (0 if singleton; 1 if the individual had a twin), maternal age and individual age (as a covariate, both linear and quadratic terms) and SNP genotypes as a three‐level factor and random effects included were maternal ID, year of birth, sheep ID (to account for the multiple observations per individual) and sheep year. The fixed effects included when analysing LR and LBS were maternal age, litter size and SNP genotypes, and the only random effects fitted were maternal ID and year of birth. A Poisson error distribution was used for AR, LR and LBS, and a categorical (binomial) error distribution was used for AS, respectively. Chains were run for 2 500 000 iterations with a burn‐in phase of 500 000 iterations, and 1000 independent samples were taken at 2000 iteration intervals. Weak priors were specified, such that the total phenotypic variance was divided equally between the random effects fitted. Results are presented as posterior modes of the 1000 sampled iterations and the 95% credibility interval (CI). Significance of effect sizes can be assumed if the 95% CI does not overlap with zero.

For each genotype at genome‐wide significant SNPs, we calculated selection coefficients for all four fitness measures as 1–*w*, where *w* stands for the relative fitness of a genotype compared to the fittest genotype. For loci and traits where heterozygote advantage was observed, we then calculated allele frequencies at equilibrium as follows:

$$q=\frac{s_{1}}{s_{1}+s_{2}}$$

where *q* is the equilibrium frequency of the minor allele, *s*_1_ is the selection coefficient of the major homozygote and *s*_2_ is the selection coefficient of the minor homozygote.

### Inference of haplotypes in QTL regions

We estimated pairwise linkage disequilibrium (LD) and inferred phased haplotypes using all SNPs found within 1 Mb either side of the most strongly associated SNPs in the Soay sheep data set. LD was calculated as the allelic correlation *r* ^2^ using the R package *LDheatmap* (Shin *et al*. 2006). To ensure optimal phasing accuracy, we phased haplotypes using all SNPs within 5 Mb either side of the most significant SNPs. Genotype data were phased, and sporadically missing SNP data were imputed using beagle v3 software (Browning & Browning 2007), which was run for 20 iterations. Twenty phased haplotype pairs were sampled from each individual. To ensure maximum comparability of the phased haplotype results with the pairwise LD results, we only used the SNPs within 1 Mb either side of the SNPs showing strongest association with trait variation in our graphical representation and extremely rare haplotypes (observations <5 which approximately corresponds to a frequency of <0.04%) were not included.

Approximately 22% of each Soay sheep's genome consists of Dunface sheep genetic material due to an admixture event in the 19th century (Feulner *et al*. 2013). Dunface sheep are now extinct, but their genetic material exists in the Boreray sheep: a breed which was created by hybridization between Dunface and Scottish Blackface sheep and which lives on another island in the St Kilda archipelago. Therefore, if a haplotype has entered the Soay population through this admixture event, haplotype sharing (HS) of core haplotypes should be greater between Soay sheep and Borerays than between Soays and other sheep breeds (including Scottish Blackface sheep).

We used our own genotype data (5805 Soay sheep) and genotype data generated by the International Sheep Genomics Consortium (ISGC) (2709 sheep belonging to 73 other domestic sheep breeds of which 17 were Boreray sheep, Table S3, Supporting information) to estimate the amount of HS between Soay sheep and other sheep breeds following the approach used by Feulner *et al*. 2013;. Quality control was performed in both data sets separately in plink v1.07 (Purcell *et al*. 2007) with the following criteria: minor allele frequency >1%, locus call rate >99% and individual call rate >95%. We then combined both QC‐ed data sets, extracted all SNPs within 5 Mb on either side of causal SNPs and again excluded all SNPs with call rate lower than 99% or MAF <1%. Haplotypes were phased using the same settings as described above, but haplotypes were phased using all SNPs within 8 Mb either side of the focal SNP. We then defined core haplotypes from the six SNPs flanking the focal SNP (the SNP showing strongest association with trait values): three SNPs upstream and two SNPs downstream of the most significant SNPs. We calculated the extent of HS using custom scripts implemented in r (Feulner *et al*. 2013) as follows: for each core haplotype *i* identified in Soay sheep with number of observations >5, and for each non‐Soay breed (or species) *j*, we extracted all chromosomes containing the core haplotype *i* from Soays and breed *j*. Subsequently, for each pair of Soay and non‐Soay chromosomes, we determined the location of the first mismatches at SNPs upstream and downstream of the core haplotype, and from this, we estimated the length of unbroken HS in base pairs. This procedure was repeated for each pairwise comparison of Soay and non‐Soay chromosomes to obtain a mean and standard deviation of HS for each core haplotype *i* between Soay sheep and breed *j*.

## Results

### Partitioning phenotypic variance across chromosomes and genomic regions

**Figure 1:**
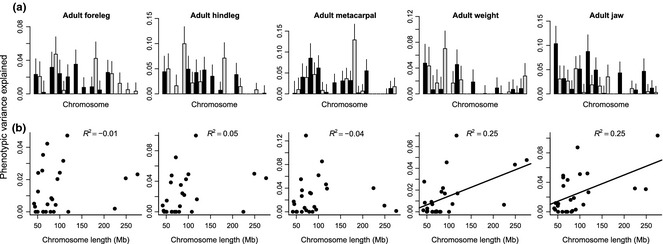
(a) The proportion of phenotypic variance for the body size traits explained by each of the 26 autosomes. (b) Scatterplot showing the correlation between the physical length of a chromosome and the phenotypic variance it explains. Solid lines are shown for linear regressions which were significant.

Genomic heritability ranged from 0.26 for foreleg to 0.53 for jaw (Table 1). When partitioning the genetic variance between chromosomes, all traits showed significant effects of individual chromosomes on phenotypic variance (Table 1, Fig. 1). Several chromosomes explained significant amounts of phenotypic variance in more than one trait, with chromosome 6 being significant for all five traits and chromosomes 16 and 19 contributing significantly to trait variance in all three leg length traits (Table 1). The sum of the point estimates of individual chromosome effects for all traits was slightly larger than the genomic heritability (Table 1). This could suggest that estimates for individual chromosomes have a tendency to be upwardly biased, but could also be due to the fact that when adding up chromosomal effect sizes, the standard errors around the estimates are not taken into account.

**Table 1:** Proportion of phenotypic variance for five body size traits explained by each chromosome. LG gives the autosome analysed, *h* ^2^ gives the proportion of phenotypic variance explained by the autosomes analysed, LRT gives the likelihood ratio test statistic. Standard errors for genetic variances are shown within parentheses

LG	Physical length (Mb)	Nr of SNP markers	Foreleg			Hindleg			Metacarpal			Weight			Jaw		
			*V*_A_	*h* ^2^	LRT	*V*_A_	*h* ^2^	LRT	*V*_A_	*h* ^2^	LRT	*V*_A_	*h* ^2^	LRT	*V*_A_	*h* ^2^	LRT
Whole genome	2452	37 037	11.107 (1.624)	0.255 (0.043)	NA	20.924 (2.91)	0.444 (0.049)	NA	7.928 (1.17)	0.494 (0.057)	NA	2.926 (0.501)	0.312 (0.046)	NA	10.69 (1.46)	0.53 (0.056)	NA
1	276	4138	1.012 (0.81)	0.023 (0.019)	2.093	2.076 (1.48)	0.044 (0.031)	2.722	0.024 (0.465)	0.001 (0.029)	0.002	0.447 (0.279)	0.048 (0.029)	3.833	2.086 (0.757)	0.103 (0.036)	**12.829**
2	249	3842	0.906 (0.765)	0.021 (0.018)	1.871	2.348 (1.436)	0.05 (0.03)	**4.103**	0.167 (0.447)	0.01 (0.028)	0.145	0.408 (0.276)	0.043 (0.029)	2.711	0.619 (0.555)	0.031 (0.027)	1.469
3	224	3540	0.085 (0.596)	0.002 (0.014)	0.022	0 (0)	0 (0)	0	0.642 (0.522)	0.04 (0.032)	1.81	0.061 (0.209)	0.006 (0.022)	0.091	0.639 (0.531)	0.032 (0.026)	1.845
4	119	1992	0 (0)	0 (0)	0	0.771 (1.016)	0.016 (0.022)	0.706	0.628 (0.436)	0.039 (0.027)	3.011	0.159 (0.195)	0.017 (0.021)	0.81	0.535 (0.465)	0.027 (0.023)	1.637
5	108	1702	1.38 (0.791)	0.032 (0.018)	**4.312**	0 (0)	0 (0)	0	1.38 (0.59)	0.085 (0.035)	**9.787**	0.067 (0.179)	0.007 (0.019)	0.132	0.196 (0.36)	0.01 (0.018)	0.371
6	117	1784	2.067 (0.895)	0.047 (0.021)	**9.933**	4.737 (1.69)	0.1 (0.034)	**17.036**	0.745 (0.462)	0.046 (0.028)	**4.175**	0.659 (0.266)	0.07 (0.027)	**14.414**	1.046 (0.515)	0.052 (0.025)	**8.126**
7	100	1653	1.056 (0.698)	0.024 (0.016)	3.492	2.316 (1.254)	0.049 (0.026)	**6.328**	0.988 (0.489)	0.062 (0.03)	**7.753**	0 (0)	0 (0)	0	1.036 (0.583)	0.051 (0.028)	**4.014**
8	91	1535	0.188 (0.476)	0.004 (0.011)	0.186	1.035 (1.019)	0.022 (0.022)	1.343	0.135 (0.316)	0.008 (0.02)	0.217	0.158 (0.172)	0.017 (0.018)	1.307	0.06 (0.348)	0.003 (0.017)	0.028
9	95	1546	0.884 (0.631)	0.02 (0.015)	2.993	2.007 (1.209)	0.043 (0.025)	**4.458**	0.061 (0.308)	0.004 (0.019)	0.037	0.426 (0.218)	0.046 (0.023)	**8.111**	1.771 (0.727)	0.087 (0.035)	**8.978**
10	86	1416	0 (0)	0 (0)	0	1.141 (1.063)	0.024 (0.022)	1.466	0 (0)	0 (0)	0	0.205 (0.202)	0.022 (0.021)	1.21	0.378 (0.404)	0.019 (0.02)	1.061
11	62	853	1.541 (0.739)	0.035 (0.017)	**8.991**	2.233 (1.231)	0.048 (0.026)	**6.206**	0.427 (0.339)	0.027 (0.021)	3.136	0 (0)	0 (0)	0	0.991 (0.524)	0.049 (0.026)	**6.375**
12	79	1218	0 (0)	0 (0)	0	0 (0)	0 (0)	0	0 (0)	0 (0)	0	0 (0)	0 (0)	0	0.327 (0.372)	0.016 (0.018)	1.032
13	83	1165	0.337 (0.513)	0.008 (0.012)	0.521	1.652 (1.084)	0.035 (0.023)	**4.283**	0.499 (0.381)	0.031 (0.024)	2.712	0.174 (0.18)	0.018 (0.019)	1.261	0.025 (0.308)	0.001 (0.015)	0.006
14	63	786	0 (0)	0 (0)	0	0 (0)	0 (0)	0	0.586 (0.413)	0.036 (0.025)	3.288	0 (0)	0 (0)	0	0.71 (0.503)	0.035 (0.025)	2.015
15	81	1204	0.37 (0.51)	0.008 (0.012)	0.646	0.345 (0.779)	0.007 (0.017)	0.248	0.516 (0.367)	0.032 (0.023)	3.396	0 (0)	0 (0)	0	0 (0)	0 (0)	0
16	72	1091	1.843 (0.849)	0.042 (0.02)	**5.959**	3.38 (1.502)	0.071 (0.031)	**7.585**	2.098 (0.66)	0.129 (0.038)	**22.814**	0.005 (0.119)	0.001 (0.013)	0.002	0 (0)	0 (0)	0
17	72	1032	0.203 (0.478)	0.005 (0.011)	0.193	0 (0)	0 (0)	0	0.042 (0.259)	0.003 (0.016)	0.032	0 (0)	0 (0)	0	0.871 (0.468)	0.043 (0.023)	**6.915**
18	69	1003	0 (0)	0 (0)	0	0 (0)	0 (0)	0	0.109 (0.257)	0.007 (0.016)	0.247	0 (0)	0 (0)	0	0 (0)	0 (0)	0
19	60	846	1.116 (0.634)	0.026 (0.015)	**6.397**	1.816 (1.092)	0.038 (0.023)	**5.25**	0.886 (0.448)	0.055 (0.027)	**8.642**	0.086 (0.151)	0.009 (0.016)	0.382	0 (0)	0 (0)	0
20	51	801	1.052 (0.633)	0.024 (0.015)	**4.639**	0.693 (0.789)	0.015 (0.017)	1.155	0 (0)	0 (0)	0	0.006 (0.114)	0.001 (0.012)	0.003	0.222 (0.322)	0.011 (0.016)	0.577
21	50	587	0 (0)	0 (0)	0	0 (0)	0 (0)	0	0 (0)	0 (0)	0	0 (0)	0 (0)	0	0.163 (0.254)	0.008 (0.013)	0.693
22	51	804	0.548 (0.549)	0.013 (0.013)	1.243	0 (0)	0 (0)	0	0 (0)	0 (0)	0	0.029 (0.145)	0.003 (0.015)	0.036	0 (0)	0 (0)	0
23	62	741	0 (0)	0 (0)	0	0 (0)	0 (0)	0	0 (0)	0 (0)	0	0.066 (0.142)	0.007 (0.015)	0.249	0.94 (0.532)	0.047 (0.026)	**4.655**
24	42	439	0.222 (0.35)	0.005 (0.008)	0.646	0.39 (0.626)	0.008 (0.013)	0.619	0.002 (0.2)	0 (0.012)	0	0.013 (0.103)	0.001 (0.011)	0.021	0.095 (0.305)	0.005 (0.015)	0.077
25	45	685	0 (0)	0 (0)	0	0 (0)	0 (0)	0	0.21 (0.282)	0.013 (0.018)	0.8	0.079 (0.143)	0.008 (0.015)	0.355	0.24 (0.322)	0.012 (0.016)	0.739
26	44	634	0.139 (0.441)	0.003 (0.01)	0.08	0.071 (0.718)	0.001 (0.015)	0.007	0.279 (0.338)	0.017 (0.021)	0.763	0.26 (0.186)	0.028 (0.02)	3.243	0.059 (0.263)	0.003 (0.013)	0.057
Sum	2452	37 037	14.948	0.343	NA	27.007	0.572	NA	10.424	0.647	NA	3.31	0.353	NA	13.009	0.645	NA

The first row shows the estimates using a GRM calculated using all SNPs and represent genomic heritability estimates. The last row shows the sum of all the individual chromosome estimates. LRT values in bold indicate that the model where a chromosome effect was fitted in addition to a polygenic effect explained significantly more of the phenotypic variation than a model only including a remaining polygenic effect.

The proportion of phenotypic variance a chromosome explained scaled with its physical length for weight and jaw (*R* ^2^ of 0.25 for both traits, *P* = 0.006 and *P* = 0.005, respectively), but this pattern was not observed for any of the leg length measures (Fig. 1). Phenotypic variance explained by a chromosome correlated significantly between some traits (e.g. between most leg length measures, weight and hindleg, and weight and jaw) but not between others (Fig. S1, Supporting information).

**Figure 2:**
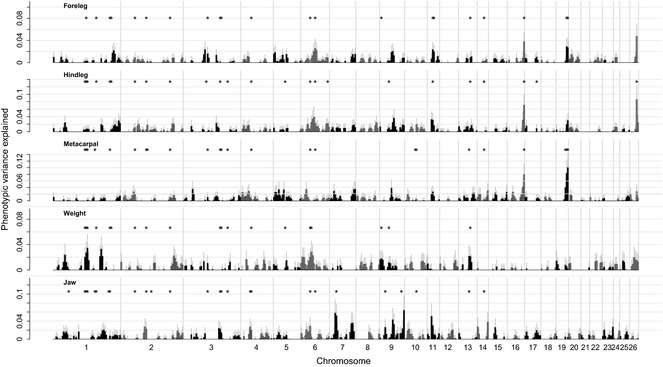
Results of regional heritability analysis of body weight in Soay sheep. Asterisks indicate regions which were significant after correcting for multiple testing.

Second, we partitioned phenotypic variance between genomic regions each containing 150 SNPs. For each trait, multiple chromosomes were found to contain regions which explained significant trait variance (Table 2, Fig. 2). Within a trait, when multiple regions on the same chromosome were found to be associated, they were often, but not always, adjacent or overlapping regions (Table 2). Several genomic regions explained significant amounts of variance in multiple traits (such as region 206 for weight and jaw, 411 and 412 for foreleg and metacarpal, Table 2), and in some cases, multiple traits were affected by neighbouring regions rather than the same region (such as regions 123 and 124 for hindleg and foreleg, respectively, Table 2). Significant regions were often but not always found on chromosomes which contributed significantly to a trait in the preceding analysis (e.g. regions on chr 6, 7, 9, 11, 13, 16, 19, Table 1, Table 2). Some regions explained significant amounts of phenotypic variance using a likelihood ratio test, but explained very little variance when fitting together with the remaining polygenic effect (Fig. 2). This could be explained by the correlation between relatedness at the region level with whole‐genome relatedness as a result of the many close relatives in the populations, which makes separating regional from polygenic effects more difficult. The contribution of regions to trait values was highly correlated between leg length traits, more modestly correlated between hindleg and weight, and weakly correlated between the remaining traits (Fig. S2, Supporting information).

**Table 2:** Results from a regional heritability analysis. Only regions which explained significant amounts of phenotypic variance are listed. Standard errors for estimates are shown between parentheses. $h_{\mathrm{Region}}^{2}$ shows the proportion of total phenotypic variations explained by regions and LRT shows the likelihood ratio test statistic. Standard errors for genetic variances are shown within parentheses. Regions highlighted in grey indicate regions which explained significant amounts of phenotypic variance in more than one trait

Trait	Region	Chromosome	Start (bp)	End (bp)	Number of SNPs	$h_{\mathrm{Region}}^{2}$	LRT	*P*
Foreleg	27	1	124 215 731	135 244 346	150	0.003 (0.006)	26.05	3.33E‐07
	35	1	163 370 112	173 759 083	150	<0.001 (<0.001)	22.369	2.25E‐06
	46	1	221 538 927	236 586 656	150	<0.001 (<0.001)	22.676	1.92E‐06
	47	1	230 555 091	241 803 266	150	0.003 (0.007)	32.136	1.44E‐08
	66	2	52 218 919	60161996	150	0.009 (0.009)	35.269	2.87E‐09
	75	2	95 657 911	106 749 240	150	<0.001 (<0.001)	19.798	8.61E‐06
	94	2	187 065 342	196 452 321	150	<0.001 (<0.001)	17.671	2.63E‐05
	124	3	88 619 302	98818184	150	0.016 (0.013)	17.214	3.34E‐05
	134	3	139 456 147	147 599 922	150	0.001 (0.004)	17.094	3.56E‐05
	135	3	143 390 505	152 762 695	150	<0.001 (<0.001)	25.497	4.43E‐07
	159	4	36 983 504	46 721 132	150	<0.001 (<0.001)	25.707	3.97E‐07
	206	6	32 615 209	43 798 415	150	<0.001 (0.003)	29.086	6.93E‐08
	210	6	53 826 107	63 013 613	150	0.025 (0.017)	26.264	2.98E‐07
	263	9	5 432 369	15 213 397	150	<0.001 (<0.001)	25.753	3.88E‐07
	304	11	23 383 949	33 722 495	150	0.024 (0.013)	19.538	9.86E‐06
	305	11	28 846 576	38 656 286	150	0.02 (0.012)	15.386	8.77E‐05
	334	13	44 268 486	55 660 662	150	0.009 (0.009)	24.59	7.09E‐07
	345	14	28 204 607	39 795 104	150	0.002 (0.004)	26.229	3.03E‐07
	377	16	64 064 879	71 555 691	116	0.038 (0.017)	29.956	4.42E‐08
	411	19	41 742 622	53 596 723	150	0.029 (0.016)	15.975	6.42E‐05
	412	19	46 920 272	58 334 807	150	0.027 (0.015)	20.034	7.61E‐06
Hindleg	26	1	119 554 142	128 404 190	150	0.005 (0.009)	28.212	1.09E‐07
	27	1	124 215 731	135 244 346	150	0.007 (0.01)	36.418	1.59E‐09
	28	1	128 490 718	139 871 327	150	0.008 (0.009)	25.839	3.71E‐07
	35	1	163 370 112	173 759 083	150	<0.001 (<0.001)	15.027	0.000106
	46	1	221 538 927	236 586 656	150	0.001 (0.007)	31.909	1.62E‐08
	47	1	230 555 091	241 803 266	150	0.006 (0.009)	37.401	9.62E‐10
	66	2	52 218 919	60 161 996	150	0.011 (0.013)	27.92	1.26E‐07
	75	2	95 657 911	106 749 240	150	0.006 (0.011)	17.73	2.55E‐05
	94	2	187 065 342	196 452 321	150	<0.001 (<0.001)	28.893	7.65E‐08
	123	3	83 846 136	94 896 073	150	<0.001 (<0.001)	27.67	1.44E‐07
	134	3	139 456 147	147 599 922	150	<0.001 (<0.001)	28.1	1.15E‐07
	140	3	166 893 525	176 707 224	150	<0.001 (<0.001)	25.718	3.95E‐07
	159	4	36 983 504	46 721 132	150	<0.001 (<0.001)	27.894	1.28E‐07
	186	5	45 779 648	55 392 813	150	<0.001 (<0.001)	19.147	1.21E‐05
	206	6	32 615 209	43 798 415	150	0.006 (0.008)	25.427	4.59E‐07
	210	6	53 826 107	63 013 613	150	0.022 (0.019)	26.651	2.44E‐07
	220	6	103 648 996	112 817 744	150	0.022 (0.014)	15.667	7.55E‐05
	269	9	32 003 055	40 498 168	150	0.011 (0.012)	18.709	1.52E‐05
	304	11	23 383 949	33 722 495	150	0.032 (0.019)	14.959	0.00011
	334	13	44 268 486	55 660 662	150	0.01 (0.012)	22.12	2.56E‐06
	345	14	28 204 607	39 795 104	150	<0.001 (<0.001)	19.572	9.69E‐06
	377	16	64 064 879	71 555 691	116	0.071 (0.029)	40.132	2.37E‐10
	387	17	47 416 116	56 753 909	150	0.009 (0.011)	17.012	3.71E‐05
	467	26	27 216 096	37 050 856	150	0.086 (0.038)	15.21	9.62E‐05
Metacarpal	26	1	119 554 142	128 404 190	150	<0.001 (<0.001)	20.984	4.63E‐06
	27	1	124 215 731	135 244 346	150	<0.001 (<0.001)	31.177	2.36E‐08
	28	1	128 490 718	139 871 327	150	0.003 (0.007)	18.866	1.40E‐05
	34	1	158 729 797	168 956 387	150	<0.001 (<0.001)	15.339	8.99E‐05
	46	1	221 538 927	236 586 656	150	<0.001 (<0.001)	25.326	4.84E‐07
	66	2	52 218 919	60 161 996	150	0.019 (0.017)	24.651	6.87E‐07
	75	2	95 657 911	106 749 240	150	<0.001 (<0.001)	20.811	5.07E‐06
	76	2	101 078 351	112 897 395	150	<0.001 (<0.001)	20.342	6.48E‐06
	94	2	187 065 342	196 452 321	150	0.01 (0.012)	24.162	8.85E‐07
	124	3	88 619 302	98 818 184	150	0.011 (0.014)	15.335	9.00E‐05
	134	3	139 456 147	147 599 922	150	<0.001 (<0.001)	16.822	4.11E‐05
	135	3	14 339 0505	152 762 695	150	<0.001 (<0.001)	26.35	2.85E‐07
	140	3	166 893 525	176 707 224	150	0.001 (0.005)	27.698	1.42E‐07
	159	4	36 983 504	46 721 132	150	0.006 (0.011)	17.979	2.23E‐05
	206	6	32 615 209	43 798 415	150	<0.001 (<0.001)	33.312	7.85E‐09
	210	6	53 826 107	63 013 613	150	0.002 (0.009)	26.059	3.31E‐07
	290	10	362 380 12	46 714 199	150	0.003 (0.007)	21.702	3.18E‐06
	291	10	41 802 553	50 290 499	150	0.005 (0.009)	22.213	2.44E‐06
	333	13	38 741 390	51 028 375	150	<0.001 (<0.001)	24.96	5.85E‐07
	345	14	28 204 607	39 795 104	150	0.004 (0.008)	26.572	2.54E‐07
	377	16	64 064 879	71 555 691	116	0.079 (0.033)	40.501	1.97E‐10
	410	19	36 615 520	46 882 636	150	0.041 (0.026)	15.437	8.53E‐05
	411	19	41 742 622	53 596 723	150	0.083 (0.037)	24.989	5.76E‐07
	412	19	46 920 272	58 334 807	150	0.101 (0.044)	32.792	1.03E‐08
Weight	26	1	119 554 142	128 404 190	150	0.021 (0.015)	22.569	2.03E‐06
	27	1	124 215 731	135 244 346	150	0.027 (0.017)	44.874	2.10E‐11
	28	1	128 490 718	139 871 327	150	0.035 (0.022)	25.181	5.22E‐07
	35	1	163 370 112	173 759 083	150	<0.001 (<0.001)	20.082	7.42E‐06
	46	1	221 538 927	236 586 656	150	<0.001 (<0.001)	22.158	2.51E‐06
	47	1	230 555 091	241 803 266	150	<0.001 (<0.001)	16.612	4.59E‐05
	66	2	52 218 919	60 161 996	150	<0.001 (<0.001)	26.075	3.28E‐07
	75	2	95 657 911	106 749 240	150	<0.001 (<0.001)	27.556	1.53E‐07
	94	2	187 065 342	196 452 321	150	<0.001 (<0.001)	24.298	8.25E‐07
	134	3	139 456 147	147 599 922	150	<0.001 (<0.001)	20.883	4.88E‐06
	135	3	143 390 505	152 762 695	150	0 (0.005)	26.129	3.19E‐07
	140	3	166 893 525	176 707 224	150	<0.001 (<0.001)	29.205	6.51E‐08
	159	4	36 983 504	46 721 132	150	<0.001 (<0.001)	23.466	1.27E‐06
	186	5	45 779 648	55 392 813	150	0.004 (0.007)	17.158	3.44E‐05
	206	6	3 261 5209	43 798 415	150	0.02 (0.014)	34.817	3.62E‐09
	207	6	38 952 950	48 762 234	150	0.028 (0.017)	15.1	0.000102
	263	9	5 432 369	15 213 397	150	0.018 (0.014)	27.856	1.31E‐07
	269	9	32 003 055	40 498 168	150	0.012 (0.01)	23.933	9.97E‐07
	334	13	44 268 486	55 660 662	150	0.021 (0.015)	25.161	5.27E‐07
Jaw	13	1	57 789 949	67 062 156	150	0.001 (0.008)	19.776	8.71E‐06
	26	1	119 554 142	128 404 190	150	<0.001 (<0.001)	22.468	2.14E‐06
	27	1	124 215 731	135 244 346	150	<0.001 (<0.001)	33.977	5.58E‐09
	28	1	128 490 718	139 871 327	150	<0.001 (<0.001)	30.246	3.81E‐08
	34	1	158 729 797	168 956 387	150	<0.001 (<0.001)	18.938	1.35E‐05
	35	1	163 370 112	173 759 083	150	<0.001 (<0.001)	32.573	1.15E‐08
	45	1	215 348 022	230 306 316	150	0 (0.006)	28.309	1.03E‐07
	46	1	221 538 927	236 586 656	150	0.007 (0.013)	32.183	1.40E‐08
	66	2	52 218 919	60 161 996	150	<0.001 (<0.001)	38.167	6.49E‐10
	75	2	95 657 911	106 749 240	150	0.022 (0.017)	38.826	4.63E‐10
	79	2	119 360 781	128 938 914	150	<0.001 (<0.001)	22.751	1.84E‐06
	94	2	187 065 342	196 452 321	150	<0.001 (<0.001)	32.984	9.29E‐09
	124	3	88 619 302	98 818 184	150	<0.001 (<0.001)	22.409	2.20E‐06
	134	3	139 456 147	147 599 922	150	0.023 (0.015)	44.695	2.30E‐11
	135	3	143 390 505	152 762 695	150	0.015 (0.013)	22.202	2.45E‐06
	140	3	166 893 525	176 707 224	150	<0.001 (<0.001)	32.509	1.19E‐08
	158	4	31 436 601	42 004 010	150	0.027 (0.018)	18.357	1.83E‐05
	159	4	36 983 504	46 721 132	150	0.02 (0.018)	26.278	2.96E‐07
	206	6	32 615 209	43 798 415	150	0.029 (0.02)	45.89	1.25E‐11
	210	6	53 826 107	63 013 613	150	<0.001 (<0.001)	36.304	1.69E‐09
	227	7	22 097 462	31 127 673	150	0.052 (0.027)	16.18	5.76E‐05
	266	9	19 630 364	27 593 399	150	0.027 (0.018)	16.272	5.49E‐05
	279	9	78 846 991	87 056 637	150	0.013 (0.014)	31.83	1.68E‐08
	291	10	41 802 553	50 290 499	150	<0.001 (<0.001)	29.369	5.98E‐08
	333	13	38 741 390	51 028 375	150	<0.001 (<0.001)	32.078	1.48E‐08
	345	14	28 204 607	39 795 104	150	<0.001 (<0.001)	37.129	1.11E‐09

### Genome‐wide association (GWAS)

**Figure 3:**
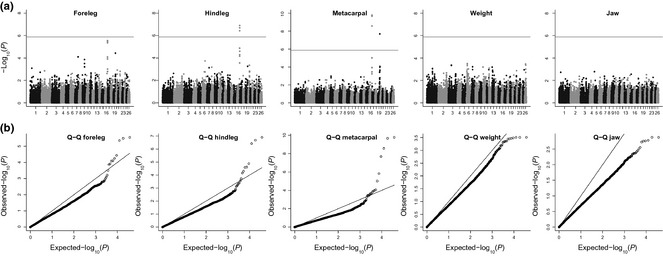
(a) genome‐wide association (GWAS) of body size traits in Soay sheep. All points above the solid line are genome‐wide significant after Bonferroni correction for multiple testing. (b) Q‐Q plot showing the observed distribution of *P* values against the expected *P* values under the null hypothesis of no association. The solid line indicates the one to one line. Generally the *P* values are below the solid line, indicating that our *P* values are deflated. From left to right, inflation factors (λ) were 0.76, 0.74, 0.82, 0.67 and 0.61. Test statistics as shown in the top panel have not been adjusted using genomic control.

After correcting for multiple testing, we found significant associations between SNP markers and hindleg and metacarpal (Table 3, Fig. 3). For both traits, the same three SNP markers on chromosome 16 exceeded genome‐wide level significance, while for metacarpal, one SNP on chromosome 19 (s74894.1) was significant. While s74894.1 was not significantly associated with either foreleg or hindleg, in both traits, it was among the SNPs showing the strongest association (Table S4, Supporting information). The three SNP markers which were significant for the two other leg length measures on chromosome 16 also approached significance for foreleg (Table 3, Fig. 3). No SNPs exceeded the genome‐wide significance threshold for either weight or jaw (Table S4, Supporting information, Fig. 3). Genomic inflation factor (λ) ranged between 0.61 and 0.82. We have not corrected for the deflated test statistics; hence, our GWAS results can be considered conservative. To obtain unbiased *P* values, we subsequently reestimated effect sizes for SNPs showing the strongest association with the leg measures in a mixed‐model GWAS in ASREML. Resulting associations were much more significant, and the SNP on chromosome 16 with the strongest signal for foreleg now exceeded genome‐wide significance (Table 4). s74894.1 on chromosome 19 was also significantly associated with foreleg, but not with hindleg (Table 4).

**Table 3:** Results of genome‐wide association for leg length traits. Association was performed on residuals extracted from a mixed model, and *P* values are deflated (Fig. 3) and thus overly conservative. For metacarpal length, results are shown for SNPs which showed significant association after Bonferroni correction (*P* values shown in bold). For hindleg, only the SNPs on chromosome 16 were genome‐wide significant, and for foreleg length, none of the associations were significantly associated after Bonferroni correction. But results for the three most associated SNPs on chromosome 16 and the single most associated SNP on chromosome 19 are shown for all leg length traits to highlight the congruence. The units for the additive effect of the minor allele are in mm

Trait	SNP	Chromosome	Position	Major allele	Minor allele	Number of observations	Additive effect of the minor allele	χ^2^	*P*
Foreleg	s63944.1	16	69 135 141	A	G	887	−0.676	22.262	2.38 × 10^−06^
	s23172.1	16	69 726 554	A	G	885	−0.673	21.788	3.04 × 10^−06^
	s22142.1	16	69 679 810	G	A	887	−0.657	21.223	4.09 × 10^−06^
	s74894.1	19	52 470 202	G	A	886	−0.405	17.352	3.11 × 10^−05^
Hindleg	s23172.1	16	69 726 554	A	G	897	−0.445	28.224	**1.08 **×** 10** ^−**07**^
	s22142.1	16	69 679 810	G	A	899	−0.432	27.274	**1.77 **×** 10** ^−**07**^
	s63944.1	16	69 135 141	A	G	899	−0.431	26.824	**2.23 **×** 10** ^−**07**^
	s74894.1	19	52 470 202	G	A	898	−0.201	12.792	3.48 × 10^−04^
Metacarpal	s23172.1	16	69 726 554	A	G	937	−1.476	40.079	**2.44 **×** 10** ^−**10**^
	s22142.1	16	69 679 810	G	A	940	−1.45	39.809	**2.80 **×** 10** ^−**10**^
	s63944.1	16	69 135 141	A	G	940	−1.366	35.336	**2.77 **×** 10** ^−**09**^
	s74894.1	19	52 470 202	G	A	939	−0.966	31.195	**2.33 **×** 10** ^−**08**^

**Table 4:** Additive effect and variance explained by SNPs showing strongest association with leg length. Estimates were obtained by fitting each SNP individually as a covariate in a fully specified animal model in asreml‐r. Associations significant after Bonferroni correction are shown in bold

Trait	SNP	Chromosome	Additive effect (mm)^a^	Mean trait value (mm)	*P*	$\sigma_{\mathrm{SNP}}^{2}$	$\sigma_{A}^{2}$	*h* ^2^	$h_{\mathrm{SNP}}^{2}$	$\left(\sigma_{\mathrm{SNP}}^{2}\right)/(\sigma_{A}^{2})$	Minor allele frequency^b^	Genotype frequencies^c^
Foreleg	s63944.1	16	−4.147 (0.583)	126.2	**2.93E‐12**	1.493	10.963	0.252	0.034	0.136	0.045	813/68/4
	s23172.1		−4.035 (0.582)		**9.64E‐12**	1.419	10.963	0.252	0.033	0.129	0.046	813/70/4
	s22142.1		−3.979 (0.578)		**1.31E‐11**	1.397	10.963	0.252	0.032	0.127	0.046	704/172/10
	s74894.1		−2.512 (0.405)		**1.00E‐09**	1.149	10.963	0.252	0.026	0.105	0.101	825/68/4
Hindleg	s23172.1	16	−5.939 (0.774)	181.1	**5.72E‐14**	3.073	20.65	0.438	0.065	0.149	0.046	826/69/4
	s22142.1		−5.931 (0.777)		**7.48E‐14**	3.054	20.65	0.438	0.065	0.148	0.045	825/70/4
	s63944.1		−5.839 (0.769)		**9.98E‐14**	3.008	20.65	0.438	0.064	0.146	0.046	715/173/10
	s74894.1		−2.557 (0.547)		3.61E‐06	1.19	20.65	0.438	0.025	0.058	0.101	851/82/4
Metacarpal	s23172.1	16	−3.727 (0.458)	81.0	**1.74E‐15**	1.21	7.81	0.487	0.075	0.155	0.046	851/85/4
	s22142.1		−3.643 (0.451)		**2.77E‐15**	1.171	7.81	0.487	0.073	0.15	0.046	851/85/4
	s63944.1		−3.427 (0.453)		**1.14E‐13**	1.02	7.81	0.487	0.064	0.131	0.045	766/165/8
	s74894.1	19	−2.438 (0.343)	81.0	**2.70E‐12**	1.082	7.81	0.487	0.067	0.139	0.101	813/68/4

a Additive effect of each minor allele

b Minor allele frequency in the entire population (5,805 individuals)

c Frequencies of major homozygote, heterozygote and minor homozygote genotypes in the adult sheep sample, respectively.

For all the leg length traits, the most significantly associated SNPs on each chromosome were characterized by relatively low frequency minor alleles (ca. 4.5% for the SNPs on Chr 16, 10.1% for those on Chr 19, Table 4). Effect sizes were large, with each copy of the ‘short‐leg’ allele conferring a 2.5–6 mm reduction in each of the three leg length measures, which is substantial compared to the mean trait values (which ranged between 80 and 181 mm).

Low‐frequency alleles conferred shorter legs, but the effect on leg length was generally additive, thus making it unlikely that the effect sizes are biased by the low frequency of homozygotes at the short‐leg allele. Across all leg length measures, allelic variation at the most significant SNP on Chr 16 (SNP s23172.1) was responsible for between 3.4% and 7.5% of phenotypic variation, thus explaining between 13% and 15% of additive genetic variance (Table 4). For metacarpal, s74894.1 explained 2.6% and 6.7% of phenotypic variance and 10.1% and 13.8% of additive genetic variance for foreleg and metacarpal, respectively (Table 4).

### Selection

There were no significant associations in either of the sexes between SNP genotypes at s23172.1 and any of the fitness measures (Table S5, Supporting information), although females carrying two copies of the minor alleles at s23172.1 had marginally nonsignificantly higher LBS than females which were homozygous for the major allele (Table S5, Supporting information). Females which were heterozygous at s74894.1 showed significantly higher LR and LBS than females which were homozygous at this locus. Females heterozygous at s74894.1 showed higher AS and AR, although this was not significant. Similarly, males heterozygous at this locus performed better than homozygotes for both long legs and short legs across most fitness measures, but the differences were not significant.

For s23172.1, *relative fitness* was consistently highest for the long‐leg homozygotes in males (Table S6, Supporting information). In females, *relative fitness* was highest for long‐leg homozygotes at s23172 for AS only, whereas short‐leg homozygote genotypes showed the highest fitness in AR, LBS and LR. For s74894.1, *relative fitness* was relatively consistently highest for heterozygotes in both sexes, with AR in females as an exception, where the short‐leg homozygote enjoyed the highest fitness (Table S6, Supporting information). Selection coefficients for other genotypes ranged between <0.01 and 0.94, although 95% confidence intervals were very broad and overlapped in all cases suggesting that there is no statistical evidence for fitness differences between genotypes. For cases where heterozygote advantage was observed, which were 7 of 8 fitness measures analysed for s74894.1, calculated equilibrium frequencies of the short‐leg allele at s74894.1 were generally higher than the observed frequency of 0.10 and ranged between 0.15 and 0.54 (Table S6), although again 95% bootstrap confidence intervals were very broad and ranged from 0 to 1.

At both loci of interest, the major long‐leg alleles increased in frequency in the population between 1990 and 2012 (s23172.1: *b* = 0.044 ± 0.014 (%/year), *R* ^2^ = 0.301, *P* = 0.004; s74894.1: *b* = 0.099 ± 0.022 (%/year), *R* ^2^ = 0.438, *P* = 0.0003; Fig. S3, Supporting information). Gene‐drop simulations (detailed information on method used in Supporting Information) showed that these increases fall within the distribution that can be generated by stochastic processes (i.e. drift) alone (one‐tailed P for s23172.1 and s74894.1, respectively: 0.378 and 0.299, Fig. S3, Supporting information).

### Linkage disequilibrium and haplotype sharing

Of the three SNPs on chromosome 16 exceeding genome‐wide significance for metacarpal, s22142.1 and s23172.1 were adjacent to one another, while s63944.1 is found ~600 kb upstream of both SNPs. However, LD analysis revealed that within a 2‐Mb segment surrounding s23172.1, these three SNPs were in extremely high LD (*r* ^2^ > 0.9, Fig. S4) and that LD between all other SNPs in the region was substantially lower. This LD pattern suggests a haplotype block around the most strongly associated SNPs, stretching from s15712.1 to s34571.1. A very different pattern was observed when looking at LD around s74894.1 on chromosome 19. In general, LD was higher in the region around s74894.1 on Chr. 19 than in the region surrounding s23172.1 on Chr 16 (mean *r* ^2^ was, respectively, 0.19 and 0.11), but s74894.1 was in relative low LD, both with neighbouring SNPs and more distant SNP (Fig. S4, Supporting information).

We next inferred haplotypes in the same regions around each leg length QTL. In the region around s23172.1 (Chr 16), 65 unique haplotypes were inferred, of which 6 contained the short‐leg G allele at s23172.1 (Fig. S5). Interestingly, all 6 of these haplotypes were identical for ~930 KB stretching from s22694.1 to 259572.1, a region which includes the three most significant SNPs for leg length (Fig. S5) suggesting that no recombination events have occurred in this region since this haplotype arose. For long‐leg haplotypes in the same region, substantially more heterogeneity was observed. In the 2‐Mb region around s74894.1 (Chr. 19), of the 37 unique haplotypes, only five harboured the short‐leg leg allele. The most common short‐leg haplotype was observed 1052 times (out of 1166 short‐leg haplotypes). In contrast with the region around s23172.1 (Chr 16), short‐leg haplotypes have undergone more recombination events around the focal SNP as they are only identical at the focal SNP and the downstream SNP (Fig. S5, Supporting information).

**Figure 4:**
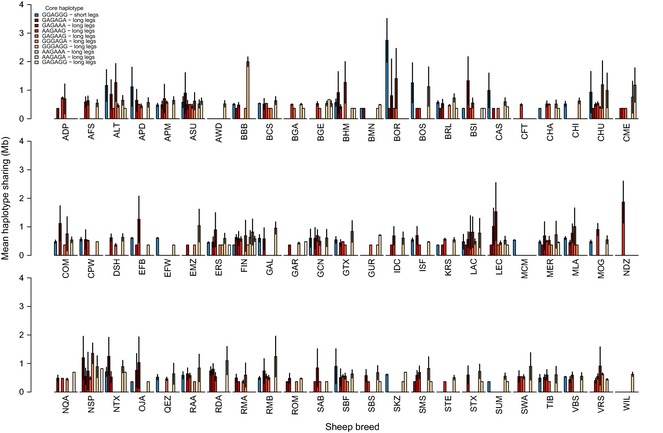
Mean and standard deviation of haplotype sharing between Soay sheep and 73 HapMap breeds for core haplotypes around s23172.1 on chromosome 16 in Soay sheep. Most relevant breed/species codes: BOR = Boreray and SBF = Scottish Blackface. All other breed codes can be found in Table S2. Haplotypes are sorted in descending order based on frequencies in the Soay sheep population. Summary statistics for haplotype sharing results can be found in Table S6.

**Figure 5:**
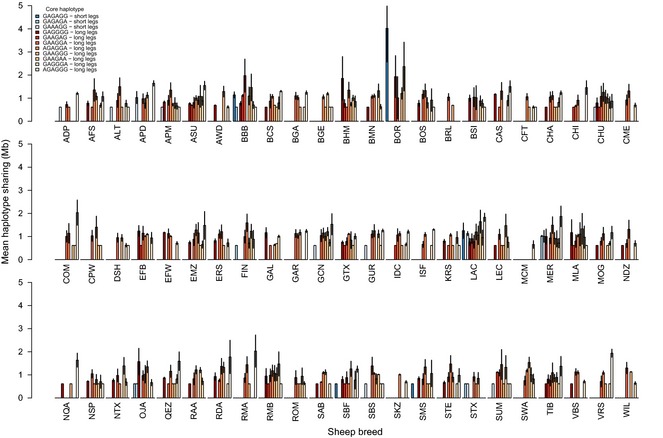
Mean and standard deviation of haplotype sharing between Soay sheep and 73 HapMap breeds for core haplotypes around s74894.1 on chromosome 19 in Soay sheep. Most relevant breed/species codes: BOR = Boreray and SBF = Scottish Blackface. All other breed codes can be found in Table S2. Haplotypes are sorted in descending order based on frequencies in the Soay sheep population. Summary statistics for haplotype sharing results can be found in Table S6.

In the Soay sheep population, we identified 10 and 11 ‘core’ haplotypes of 6 SNPs with more than five observations (covering 321Kb and 555Kb) around SNPs s23172.1 (Chr 16, Fig. 4) and s74894.1 (Chr 19, Fig. 5), respectively (Table S7). Of these unique core haplotypes, one haplotype on chromosome 16 and three haplotypes on chromosome 19 tagged short‐leg alleles. All core haplotypes found in Soay sheep were present in other sheep breeds used in the analysis. However, both the number of core haplotypes found in other sheep breeds and the length of the haplotypes surrounding the core haplotypes shared between the Soay sheep and other breeds varied substantially, reflecting that some breeds are more closely related to the Soays than others (Figs 4 and 5). For example, for several core haplotypes (haplotypes 1 and 5 at chromosome 16, haplotypes 1, 5 and 9 at chromosome 19, Table S7), HS between the Boreray sheep and Soay sheep was greater than between any other sheep breed and Soays. For both chromosomes, this pattern was particularly striking for core haplotypes carrying the minor short‐leg alleles. In fact, the extent of HS around SNPs s23172.1 (Chr 16) and s74894.1 (Chr 19) with the Borerays at the most common short‐leg haplotypes exceeded HS at all other haplotypes and with all other breeds.

## Discussion

### Heterogeneity of genetic architecture of body size

Our analysis has enhanced our understanding of the genetic architecture of body size in two ways. First, different proxies of body size showed different patterns of correlation between chromosome length and the proportion of variance explained by a chromosome (Fig. 1B). The significant positive correlations for weight and jaw indicate that many loci having small effects on these traits are scattered throughout the genome. In the three leg length traits, the expected correlation was disrupted (and not significant). While this is partly explained because two small chromosomes, 16 and 19, explained disproportionately large amounts of variation, regressions excluding those two chromosomes were still not significant. While the regional heritability analysis for all traits located numerous regions of interest scattered throughout the genome, GWAS located QTL only for the leg length measures, and the QTL were located on chromosomes 16 and 19. Thus, different aspects of a single syndrome (body size), as well as different traits, can show different genetic architectures, and assuming enough power in terms of individuals and markers (relative to LD), chromosomal partitioning is a useful tool for inferring such differences and potentially for predicting which chromosomes harbour major QTL.

Second, our analyses provide support for and illuminate the previously documented genetic correlations between body size traits (Bérénos *et al*. 2014). All five traits analysed are genetically correlated to some degree [at least 0.3 (Bérénos *et al*. 2014)], and consistent with this, we found that regions or chromosomes which explained large amounts of variation in one trait often also explained large amounts of variation in other traits. Thus, some chromosomes and even 150 SNP regions contributed significantly to both leg length and either jaw or weight, with chromosome 6 even being significant for all five traits (Table 1). However, detailed comparisons of particular pairs of traits also illuminated the variation in genetic correlations between specific traits. We would expect the different leg length measures to be under similar genetic control due to their common developmental path. And indeed, the three leg length traits showed a very consistent pattern of which genomic segments explained phenotypic variance at all of the levels of resolution analysed (chromosome, 150‐SNP region and even SNP level), confirming the strong genetic correlations between them [Table S1, Supporting information, (Bérénos *et al*. 2014)]. Many chromosomes were significant for at least two leg length measures and some even for all three (chromosomes 6, 16 and 19). Multiple regions on both chromosomes 16 and 19 explained significant amounts of trait variance using regional heritability analysis in both foreleg and metacarpal, although only chromosome 16 regions were significant for hindleg in this analysis. Two SNPs found within these regions on Chr 16 and Chr 19 were associated with foreleg and metacarpal, one of which was confirmed in hindleg. In contrast, there was much less consistency when comparing body weight, jaw and either of these traits with the leg length measures. The regional heritability analysis showed relatively low overlap of significant regions between body weight and jaw (genetic correlation ca. 0.5 (Bérénos *et al*. 2014), with just one region, 206 on chromosome 6, in common. Similarly, there was little overlap of significant regions between either weight or jaw and any of the leg length measures [genetic correlations ca. 0.3–0.5 (Bérénos *et al*. 2014)]. These contrasts were also supported by the fact that the variance explained by either whole chromosomes or regions was strongly correlated between leg length measures but only weakly correlated between weight and jaw (Figs S1 and S2, Supporting information). Estimation of genetic correlations per chromosome would have been desirable, but we lacked the power to conduct this analysis.

Absence of a positive correlation between chromosome length and genetic variance explained, such as we observed for leg length measures, is often interpreted as evidence against the polygenic model or in support of a moderate to large effect QTL model (Robinson *et al*. 2013). However, we believe, for the reasons outlined below, that the polygenic model still holds for the leg length traits. First, multiple chromosomes and regions within chromosomes explain significant amounts of genetic variance for all the leg length traits, although some chromosomes contributed to disproportionally large amounts of variance (e.g. Chr 16 and 19), and others explaining disproportionally small amounts of variance (e.g. Chr 1, 2 and 3). Disproportionally large effects of a chromosome could arise due to it carrying many genes of small effect or because of a few major effect QTL. We here show that, for both chromosome 16 and 19, most of the chromosomal heritability is explained by a single SNP. But given that the minor alleles are each almost exclusively found on a single long‐ranging haplotype, we cannot rule out the possibility that both these main effect QTL are tagging several causal SNPs or genes. Second, each of the genome‐wide significant SNPs only explain ca. 14% of the total genomic heritability for each trait, suggesting that although leg length is influenced by two major‐effect QTL, the majority of the genetic variance is explained by undiscovered variants.

Many QTL analyses in natural populations are underpowered and hence only detect major effect QTL (Slate 2013), and for the same reasons estimated inflated effect sizes (Beavis 1994). Our results are in contrast with many previous QTL studies in natural populations (which used variance component‐based linkage mapping), as the SNPs significant for hindleg length reported here only explain between 3 and 8% of phenotypic variation. This may be partly a result of the larger sample sizes used here than in many other QTL studies in wild population, leading to more realistic estimates of effect sizes (Slate 2013). An alternative explanation is that the high density of SNP markers used in GWAS methods, such as used here, only picks up an association with markers which are in LD with causal variants, whereas QTL detected using linkage mapping often cover tens of cM and may be the result of multiple linked causal loci (Slate *et al*. 2010).

The estimates of genetic variance explained by a genomic region are highly consistent between the three complementary analyses undertaken (e.g. 7% of phenotypic variance for hindleg is explained by s23172.1, region 377 and chromosome 16, and between 5.5% and 8.3% of phenotypic variance for metacarpal is explained by s74894.1, region 411 and chromosome 19, Tables 1, 2, 3). While this suggests that our single SNP effects are unlikely to be greatly inflated, the strong LD and haplotype structure around s23172.1 will make fine mapping of true causal variants very challenging. Haplotypes with minor short‐leg alleles at s23172.1 virtually always carry minor alleles at the remaining two Chr. 16 SNPs that exceed genome‐wide significance, while the opposite is true for haplotypes carrying the major long‐leg alleles. In combination with the fact that haplotypes carrying minor alleles at s23172.1 (Chr. 16) are completely identical for a region spanning almost 1 Mb, we cannot rule out that: (i) s23172.1 may tag more than one causal variant, and/or (ii) causal variants are located anywhere in this region. Perhaps the greater resolution of the newly available 600K HD Ovine SNP chip will lead to greater precision in mapping the locus underlying leg length on chromosome 16.

Despite substantially increased sample sizes, we were only partly able to replicate earlier findings obtained in this study population using variance component‐based linkage mapping, in which a significant QTL for jaw was found on chromosome 11, and suggestive QTL on chromosomes 15 and 8 for hindleg and birthweight, respectively (Beraldi *et al*. 2007). We found that chromosome 11 did explain significant variation for three traits, including jaw, while a region on this chromosome was found to explain large amounts of variation in foreleg and hindleg in regional heritability analyses (Fig. 1 and Fig. 2, Table 1 and Table 2). However, we were not able to replicate previous findings using GWAS. This may be due to the previous study suffering more from the aforementioned Beavis effect (Slate 2013). Also, major effect QTL found using a linkage mapping approach could be a result of the effect of multiple linked loci, each with a small effect of its own, and such QTL are less likely to be confirmed using a single‐SNP GWAS approach. Methods relying on partitioning variance between chromosomes or genomic regions are better suited to detect such joint effects of linked loci each with an effect too small to be detected by GWAS (Nagamine *et al*. 2012), thus explaining why a QTL previously found using the variance component approach was only replicated using chromosome partitioning. Our results are also in agreement with results in the only study we are aware of which examined the genetic architecture of weight using the OvineSNP50 chip in a domestic sheep breed. In this study, it was shown that regions on chromosome 6 affect body weight in Blackface sheep lambs (Riggio *et al*. 2013). These overlap with regions on chromosome 6 that explain significant phenotypic variance in both weight and jaw in Soay sheep in this study.

### Can polymorphism at leg length QTL be traced to admixture with Dunface sheep?

Recently, it has been shown that Soay sheep have experienced an admixture event with the Dunface sheep breed and that this admixture event introduced alleles and discrete phenotypic variation for coat colour and colour pattern (Feulner *et al*. 2013). Although Dunface sheep are now extinct, their genetic material persists in the Boreray sheep, which were created by admixture between Dunface and Blackface sheep. Here, we demonstrate that phenotypic and genetic variation in a quantitative trait probably also originated through the admixture event with this more modern breed described in (Feulner *et al*. 2013). The extensive HS with Boreray sheep of the short‐leg allele haplotypes on both chromosomes 16 and 19, and low number of breeds in which core haplotypes carrying the short‐leg allele at s74894.1 were found suggest that these alleles have been introduced from the Dunface sheep. In comparison, HS of haplotypes tagging the long‐leg alleles with Borerays and other breeds was comparatively much lower, indicating that more recombination events have occurred as the Soay sheep diverged from other sheep breeds and thus that long‐leg haplotypes were present in the Soay sheep population prior to the admixture event with Dunface sheep. Given that the introgressed alleles account for a reasonable proportion of the genetic variance for leg length, our results support the recent reappraisal of the contribution of interpopulation or interspecific hybridization to evolution in natural populations (Green *et al*. 2010; Salazar *et al*. 2010; Staubach *et al*. 2012).

The combined frequencies of the haplotypes tagging the short‐leg alleles (region on chr. 16: 4.6%, region on chr. 19: 10.1%) are lower than the proportion of the Soay sheep genome which is estimated to be derived from the admixture event (22%, Feulner *et al*. 2013). One explanation is that some of the long‐leg haplotypes present in the Soay sheep may have also been present in the Dunface sheep. This is supported by the observation that the amount of HS for several ‘long‐leg’ haplotypes with the Boreray sheep, although low, still exceeded that with all other sheep breeds. A second explanation is that the frequency of short‐leg haplotypes has decreased since the admixture event. Indeed, we show that allele frequencies of minor short‐leg alleles have declined over the duration of the study period (Fig. S3, Supporting information), although it would be unwise to extrapolate this negative trend prior to 1990.

Admixture is ubiquitous, as few populations exist in continuous isolation. It is probably on the rise due to human‐facilitated movement of organisms, and our consideration of its importance as an evolutionary force is growing (Grant *et al*. 2004). Novel genetic variants are often introduced at higher frequencies as a consequence of admixture than when they arise through mutations (Hedrick 2013) and have been exposed to natural selection in previous environments which increases the chance that they are advantageous (Barrett & Schluter 2007), thereby potentially leading to increased power to detect associations between traits and loci. Furthermore, our results support previous studies which have shown that introgression from domesticated populations can introduce new genetic variants which have presumably arisen during artificial selection (Anderson *et al*. 2009).

### Are there fitness differences between leg length QTL?

Body size is often found to be under positive selection in natural populations (Kingsolver & Pfennig 2004). In Soay sheep, body size covaries positively with fitness at the phenotypic level, but using quantitative genetic tools, it was previously shown that this covariance is explained by environmental covariance rather than genetic covariance (Morrissey *et al*. 2012) and hence that a genetic response to selection is unlikely. Consistent with this, we show that when looking at raw fitness measures, without controlling for potentially confounding environmental variables, genotypes at SNPs which are significantly associated with leg length do not differ significantly in annual survival, annual reproductive success, LBS or lifetime number of recruits (LR). There was a tendency for individuals heterozygous at s74894.1 to show greatest relative fitness in 7 of the 8 fitness measures, but this difference was not significant. However, differences in fitness can be explained by factors independent of leg length loci, such as maternal age, and whether or not an individual had a twin sibling, which may be confounded and thus potentially mask underlying fitness differences between genotypes. When we included those variables in our models, we found that indeed females heterozygous at s74894.1 produced significantly more lambs and recruits than females homozygous for the long‐leg allele (Table S5, Supporting information). While not significant, this was seen consistently for all other sex‐specific fitness measures.

While the results from the more elaborate models are in broad agreement with the relative fitness estimates and selection coefficients on the genotypes, there were, as discussed in the previous paragraph, differences in the statistical support. However, the similarity in sign between both sets of analyses seems to suggest that there is possibly evidence for heterozygote advantage at the genotype level, but that as fitness differences between individuals can arise due to nongenetic factors, this advantage is partly masked by other processes such as differences in environment or maternal age differences. Empirical examples of overdominance are rare (Allison 1954), and one of the few examples of heterozygote advantage in a free‐living populations was previously found in our study population, where overdominance at the locus underlying horn type and horn size (*RXFP2*) was observed in males (Johnston *et al*. 2013).

However, we should emphasize that our results should not be interpreted as evidence for heterozygote advantage. First, whereas females heterozygous for s74894.1 showed higher fitness than long‐leg homozygote females, 95% credibility intervals overlapped with fitness estimates for the ‘short‐leg’ homozygote females. The large CI are most likely the result of the extremely small sample sizes for the latter genotype. Thus, while this study, like many studies examining selection on genetic loci in wild populations, is underpowered, the low minor allele frequencies at both loci lead to even further reduction in power and precision, which is in our case especially noticeable for the short‐leg homozygotes. Hence, the only conclusion we can make based on our results is that there is evidence for a fitness advantage of the short‐leg allele at s74894.1, which could arise due to heterozygote advantage, or a (partial) dominant or even additive effect of the short‐leg allele on fitness.

Interestingly, the frequencies of alleles associated with short stature have declined over the study period. We have shown that these changes can be sufficiently explained by stochastic processes alone, suggesting that there is no response to directional selection at either leg length locus. For s74894.1, the sign of allelic change is opposite to what we would expect based on the equilibrium frequencies, as currently observed short‐leg allele frequencies at s74894.1 are much lower than expected (Table S6, Supporting information). However, these two observations are not necessarily in disagreement, as the confidence intervals around the equilibrium frequencies span the entire range from 0 to 1 which is probably the result of the extremely small sample sizes for the homozygous short‐leg genotypes.

The lack of detectable directional selection on SNPs which were significantly associated with hindleg suggests that introgressed Dunface haplotypes did not interact negatively with the Soay sheep genetic background. Our results are thus consistent with the lack of negative fitness consequences of introgressed haplotypes carrying causal loci affecting coat colour in Soay sheep (Gratten *et al*. 2012; Feulner *et al*. 2013).

In summary, we here show that different proxies of body size in Soay sheep are influenced by different genomic regions and that the degree of overlap broadly corresponds with the strength of the genetic correlation between the traits. While two body size traits (jaw and weight) probably adhere to the infinitesimal model, the leg length traits are influenced by two loci with moderately large effect. Interestingly, genetic polymorphism in both regions has probably arisen as a result of admixture, but we have no evidence that the introduced alleles are selected against. If anything, it is possible that females carrying ‘short‐leg’‐introduced alleles have a higher fitness than females carrying ‘long‐leg’ alleles at one of the loci underlying leg length. Our results demonstrate that in combination with detailed phenotype and fitness information, dense marker panels, which are increasingly available for model and nonmodel systems, can be powerful tools to unravel the genetic architecture, the origin of genetic variance for complex traits in natural populations, and lead to deeper insights into selection operating on those traits than can be obtained using pedigrees alone.

J.G.P and J.M.P. organised the long‐term collection of phenotypic data and DNA samples, C.B. and P.A.E. performed laboratory work, S.H.L. analysed data from a pilot study, J.G. wrote custom R scripts for the HS and gene‐dropping analyses, C.B analysed the data, C.B and J.M.P wrote the manuscript, all authors have approved the final version.

## Data accessibility

SNP genotype data for all phenotyped individuals, SNP Genotype info for individuals included in the fitness analyses, SNP genotype information for loci used in the haplotype sharing analyses, body size measures, fitness data, fixed and random effect information used in all analyses can be found on Dryad: doi:10.5061/dryad.b6s6q.

## Supporting information


**Table S1** Estimates of genetic covariances and correlations between adult body size traits.
**Table S2** List of 150 SNP windows used in the regional heritability analysis.
**Table S3** List of domestic sheep breeds used in the haplotype‐sharing analyses.
**Table S4** List of the 15 SNPs showing strongest associations using GWAS for each trait.
**Table S5** Parameter estimates for fixed effects from sex‐specific Bayesian models of annual survival, number of annual recruits, lifetime breeding success (LBS) and lifetime number of recruits (LR).
**Table S6** Selection coefficients for sex‐specific annual survival, annual recruitment, lifetime breeding success (LBS) and lifetime number of recruits (LR) at genotypes at two SNPs which were genome‐wide significant for leg length.
**Table S7** The extent of haplotype sharing between Soays and domestic sheep breeds around genome‐wide significant SNPs for hindleg length on chromosomes 16 and 19.
**Fig S1** Correlation of variance explained by each chromosome between traits.
**Fig S2** Correlation of variance explained by each 150 SNP window between traits.
**Fig S3** Population frequency of (A) s23172.1 and (C) s74894.1 in Village Bay between 1990 and 2012.
**Fig S4** Heatmaps of pairwise LD between SNPs within 1 MB either side of the SNPs on chromosome 16 and 19 showing strongest association with metacarpal length.
**Fig S5** Unique haplotypes in the Soay sheep population in a region within 1 MB either side of s23172.1 on chromosome 16 and s74894.1 on chromosome 19 showing strongest association with metacarpal length.
