# Supplementary material for: Heterogeneity of genetic architecture of body size traits in a free‐living population
Source: Mol Ecol. 2015 Mar 30;24(8):1810–30. doi: 10.1111/mec.13146 (PMC4405094; doi:10.1111/mec.13146)
Supplement: Supplementary file 1 — Table S1 Estimates of genetic covariances and correlations between adult body size traits. Table S2 List of 150 SNP windows used in the regional heritability analysis. Table S3 List of domestic sheep breeds used in the haplotype‐sharing analyses. Table S4 List of the 15 SNPs showing strongest associations using GWAS for each trait. Table S5 Parameter estimates for fixed effects from sex‐specific Bayesian models of annual survival, number of annual recruits, lifetime breeding success (LBS) and lifetime number of recruits (LR). Table S6 Selection coefficients for sex‐specific annual survival, annual recruitment, lifetime breeding success (LBS) and lifetime number of recruits (LR) at genotypes at two SNPs which were genome‐wide significant for leg length. Table S7 The extent of haplotype sharing between Soays and domestic sheep breeds around genome‐wide significant SNPs for hindleg length on chromosomes 16 and 19. Fig S1 Correlation of variance explained by each chromosome between traits. Fig S2 Correlation of variance explained by each 150 SNP window between traits. Fig S3 Population frequency of (A) s23172.1 and (C) s74894.1 in Village Bay between 1990 and 2012. Fig S4 Heatmaps of pairwise LD between SNPs within 1 MB either side of the SNPs on chromosome 16 and 19 showing strongest association with metacarpal length. Fig S5 Unique haplotypes in the Soay sheep population in a region within 1 MB either side of s23172.1 on chromosome 16 and s74894.1 on chromosome 19 showing strongest association with metacarpal length. [file mec0024-1810-sd1.docx]

**Supporting methods**

*Gene- drop analysis*

We analysed temporal trends in allele frequencies at genome-wide significant SNPs in the Village Bay study population between 1990 and 2012 using linear regression. Temporal trends in allele frequencies can occur as a result of stochastic processes such as genetic drift alone, hence we tested whether the slope of the temporal trend was steeper than expected based on drift alone using gene dropping simulations ([Gratten *et al.* 2012](#_ENREF_2)). This approach accounts for known pedigree structure and fluctuations in population size by randomly dropping alleles at genome-wide significant SNPs through the pedigree.

At the start of the pedigree, founders were randomly assigned genotypes based on the allele frequencies in animals alive in that year. Alleles were randomly sampled from each parent and dropped to their offspring born the following year. Gene-dropping was done on a per-cohort basis, such that in each year, paternal or maternal alleles of non-founders were randomly dropped to their offspring. One of the features of wild populations is that immigrants appear throughout the pedigree and thus maternal or paternal links are not known for all individuals. Hence, for each cohort allele frequencies were re-estimated, and immigrants were randomly assigned genotypes based on allele frequencies in the previous year. Gene-dropping simulations were repeated 1000 times for each cohort between 1990 and 2012. From each simulation we calculated the slope of the linear regression for the major allele frequencies at loci of interest between 1990 and 2012. The relative importance of genetic drift and directional selection was assessed by comparing the observed regression slope with the distribution of 1000 simulated slopes. Directional selection can only be inferred if the observed slope falls outside the 95% confidence interval of the simulated slopes.

**Table S1:** Estimates of genetic covariances and correlations between adult body size traits. Estimates are shown as means with standard errors within parentheses. Estimates are taken from a previous paper, and the estimates were obtained using animal models fitting a genomic relatedness matrix ([Bérénos *et al.* 2014](#_ENREF_1)).

| Trait combination | Additive genetic covariance | Additive genetic correlation |
| --- | --- | --- |
| Foreleg-Weight | 2.347 (0.713) | 0.446 (0.097) |
| Hindleg-Weight | 3.158 (0.968) | 0.455 (0.095) |
| Hindleg-Foreleg | 13.65 (2.1) | 0.944 (0.019) |
| Hindleg-Metacarpal | 11.669 (1.671) | 0.889 (0.041) |
| Foreleg-Metacarpal | 8.362 (1.193) | 0.91 (0.038) |
| Weight-Metacarpal | 1.436 (0.63) | 0.292 (0.117) |
| Hindleg-Jaw | 6.917 (1.716) | 0.492 (0.085) |
| Foreleg-Jaw | 4.427 (1.229) | 0.442 (0.094) |
| Weight-Jaw | 2.862 (0.706) | 0.545 (0.097) |
| Metacarpal -Jaw | 3.883 (1.182) | 0.414 (0.102) |

**Table S2:** List of 150 SNP windows used in the regional heritability analysis.

| **Region** | **Chromosome** | **Position of first SNP** | **Position of last SNP** | **Number of SNPs**  **in region** |
| --- | --- | --- | --- | --- |
| 1 | 1 | 52854 | 9225672 | 150 |
| 2 | 1 | 4951877 | 13716153 | 150 |
| 3 | 1 | 9241769 | 18695502 | 150 |
| 4 | 1 | 13761431 | 23651580 | 150 |
| 5 | 1 | 18773114 | 28706320 | 150 |
| 6 | 1 | 23709900 | 32871895 | 150 |
| 7 | 1 | 28748336 | 38056915 | 150 |
| 8 | 1 | 32915213 | 41997167 | 150 |
| 9 | 1 | 38095589 | 46818281 | 150 |
| 10 | 1 | 42108474 | 52484108 | 150 |
| 11 | 1 | 46956837 | 57783473 | 150 |
| 12 | 1 | 52559137 | 62660714 | 150 |
| 13 | 1 | 57789949 | 67062156 | 150 |
| 14 | 1 | 62731658 | 71535484 | 150 |
| 15 | 1 | 67105217 | 75595951 | 150 |
| 16 | 1 | 71578681 | 79539160 | 150 |
| 17 | 1 | 75639963 | 84871417 | 150 |
| 18 | 1 | 79569865 | 89286287 | 150 |
| 19 | 1 | 84970296 | 94332471 | 150 |
| 20 | 1 | 89419206 | 99024896 | 150 |
| 21 | 1 | 94466756 | 104053989 | 150 |
| 22 | 1 | 99077958 | 110289920 | 150 |
| 23 | 1 | 104095671 | 114766836 | 150 |
| 24 | 1 | 110359176 | 119491972 | 150 |
| 25 | 1 | 114785821 | 124183658 | 150 |
| 26 | 1 | 119554142 | 128404190 | 150 |
| 27 | 1 | 124215731 | 135244346 | 150 |
| 28 | 1 | 128490718 | 139871327 | 150 |
| 29 | 1 | 135388169 | 144607781 | 150 |
| 30 | 1 | 139926856 | 150396918 | 150 |
| 31 | 1 | 144626184 | 154793635 | 150 |
| 32 | 1 | 150543477 | 158711292 | 150 |
| 33 | 1 | 154814819 | 163257303 | 150 |
| 34 | 1 | 158729797 | 168956387 | 150 |
| 35 | 1 | 163370112 | 173759083 | 150 |
| 36 | 1 | 169008412 | 179190092 | 150 |
| 37 | 1 | 173862929 | 184155995 | 150 |
| 38 | 1 | 179234730 | 189356267 | 150 |
| 39 | 1 | 184199220 | 193817503 | 150 |
| 40 | 1 | 189368321 | 197634146 | 150 |
| 41 | 1 | 193847444 | 202516622 | 150 |
| 42 | 1 | 197694898 | 207719161 | 150 |
| 43 | 1 | 202580942 | 214975344 | 150 |
| 44 | 1 | 207785101 | 221464582 | 150 |
| 45 | 1 | 215348022 | 230306316 | 150 |
| 46 | 1 | 221538927 | 236586656 | 150 |
| 47 | 1 | 230555091 | 241803266 | 150 |
| 48 | 1 | 236639011 | 246869492 | 150 |
| 49 | 1 | 241820197 | 251959389 | 150 |
| 50 | 1 | 246874790 | 256682640 | 150 |
| 51 | 1 | 252011997 | 260791136 | 150 |
| 52 | 1 | 256714494 | 265385121 | 150 |
| 53 | 1 | 260797484 | 270423980 | 150 |
| 54 | 1 | 265440987 | 274740631 | 150 |
| 55 | 2 | 158066 | 9392243 | 150 |
| 56 | 2 | 5011935 | 13824172 | 150 |
| 57 | 2 | 9434535 | 17969084 | 150 |
| 58 | 2 | 13836112 | 22187452 | 150 |
| 59 | 2 | 18001962 | 26263538 | 150 |
| 60 | 2 | 22316785 | 32211152 | 150 |
| 61 | 2 | 26300198 | 36709532 | 150 |
| 62 | 2 | 32251867 | 41567516 | 150 |
| 63 | 2 | 36763273 | 46598180 | 150 |
| 64 | 2 | 41614741 | 52177597 | 150 |
| 65 | 2 | 46648569 | 56556228 | 150 |
| 66 | 2 | 52218919 | 60161996 | 150 |
| 67 | 2 | 56639600 | 65017658 | 150 |
| 68 | 2 | 60252367 | 70160330 | 150 |
| 69 | 2 | 65040471 | 75142623 | 150 |
| 70 | 2 | 70246423 | 80811295 | 150 |
| 71 | 2 | 75148613 | 85724528 | 150 |
| 72 | 2 | 80857803 | 90977972 | 150 |
| 73 | 2 | 85852779 | 95621490 | 150 |
| 74 | 2 | 91015456 | 101030254 | 150 |
| 75 | 2 | 95657911 | 106749240 | 150 |
| 76 | 2 | 101078351 | 112897395 | 150 |
| 77 | 2 | 106830369 | 119340905 | 150 |
| 78 | 2 | 113014357 | 124443428 | 150 |
| 79 | 2 | 119360781 | 128938914 | 150 |
| 80 | 2 | 124467465 | 133928107 | 150 |
| 81 | 2 | 129018506 | 138813572 | 150 |
| 82 | 2 | 133942420 | 143259398 | 150 |
| 83 | 2 | 138871425 | 147448621 | 150 |
| 84 | 2 | 143297335 | 151870611 | 150 |
| 85 | 2 | 147583354 | 155927328 | 150 |
| 86 | 2 | 151888029 | 160729558 | 150 |
| 87 | 2 | 155969590 | 164845468 | 150 |
| 88 | 2 | 160748450 | 169129216 | 150 |
| 89 | 2 | 164930370 | 172917684 | 150 |
| 90 | 2 | 169169208 | 177352101 | 150 |
| 91 | 2 | 173034908 | 182124730 | 150 |
| 92 | 2 | 177439223 | 186972217 | 150 |
| 93 | 2 | 182159410 | 191650061 | 150 |
| 94 | 2 | 187065342 | 196452321 | 150 |
| 95 | 2 | 191673164 | 201039949 | 150 |
| 96 | 2 | 196487903 | 205100867 | 150 |
| 97 | 2 | 201064060 | 210115127 | 150 |
| 98 | 2 | 205223167 | 215498705 | 150 |
| 99 | 2 | 210156039 | 221892049 | 150 |
| 100 | 2 | 215545833 | 226196223 | 150 |
| 101 | 2 | 221940916 | 231340317 | 150 |
| 102 | 2 | 226251202 | 235917576 | 150 |
| 103 | 2 | 231407490 | 242738945 | 150 |
| 104 | 2 | 235922123 | 247922953 | 150 |
| 105 | 3 | 143455 | 10515273 | 150 |
| 106 | 3 | 5668047 | 16116866 | 150 |
| 107 | 3 | 10540976 | 20116222 | 150 |
| 108 | 3 | 16189589 | 24062237 | 150 |
| 109 | 3 | 20204035 | 27923071 | 150 |
| 110 | 3 | 24093757 | 32358316 | 150 |
| 111 | 3 | 27979457 | 36296085 | 150 |
| 112 | 3 | 32423717 | 40781801 | 150 |
| 113 | 3 | 36351435 | 46053225 | 150 |
| 114 | 3 | 40849521 | 50657584 | 150 |
| 115 | 3 | 46079751 | 55916023 | 150 |
| 116 | 3 | 50713075 | 61051521 | 150 |
| 117 | 3 | 55962361 | 65256677 | 150 |
| 118 | 3 | 61105649 | 70340084 | 150 |
| 119 | 3 | 65290018 | 74530499 | 150 |
| 120 | 3 | 70381165 | 79138436 | 150 |
| 121 | 3 | 74725586 | 83805632 | 150 |
| 122 | 3 | 79205761 | 88596605 | 150 |
| 123 | 3 | 83846136 | 94896073 | 150 |
| 124 | 3 | 88619302 | 98818184 | 150 |
| 125 | 3 | 94923033 | 104217317 | 150 |
| 126 | 3 | 98909201 | 109608746 | 150 |
| 127 | 3 | 104352977 | 113495898 | 150 |
| 128 | 3 | 109665677 | 119127051 | 150 |
| 129 | 3 | 113589253 | 125054701 | 150 |
| 130 | 3 | 119159787 | 130068556 | 150 |
| 131 | 3 | 125101406 | 134276931 | 150 |
| 132 | 3 | 130103393 | 139367564 | 150 |
| 133 | 3 | 134338764 | 143335973 | 150 |
| 134 | 3 | 139456147 | 147599922 | 150 |
| 135 | 3 | 143390505 | 152762695 | 150 |
| 136 | 3 | 147790435 | 157153421 | 150 |
| 137 | 3 | 152855288 | 160758526 | 150 |
| 138 | 3 | 157161446 | 166799096 | 150 |
| 139 | 3 | 160802517 | 171544143 | 150 |
| 140 | 3 | 166893525 | 176707224 | 150 |
| 141 | 3 | 171572613 | 181006032 | 150 |
| 142 | 3 | 176744641 | 185471518 | 150 |
| 143 | 3 | 181055903 | 190881196 | 150 |
| 144 | 3 | 185498579 | 195014481 | 150 |
| 145 | 3 | 190907829 | 200016587 | 150 |
| 146 | 3 | 195048480 | 204596053 | 150 |
| 147 | 3 | 200031464 | 209633086 | 150 |
| 148 | 3 | 204915080 | 213524510 | 150 |
| 149 | 3 | 209736128 | 218012025 | 150 |
| 150 | 3 | 213625709 | 222747485 | 150 |
| 151 | 4 | 235989 | 10206259 | 150 |
| 152 | 4 | 5282504 | 14899633 | 150 |
| 153 | 4 | 10281638 | 19127280 | 150 |
| 154 | 4 | 14933925 | 23272098 | 150 |
| 155 | 4 | 19207285 | 27330051 | 150 |
| 156 | 4 | 23339214 | 31330763 | 150 |
| 157 | 4 | 27390887 | 36923289 | 150 |
| 158 | 4 | 31436601 | 42004010 | 150 |
| 159 | 4 | 36983504 | 46721132 | 150 |
| 160 | 4 | 42065176 | 50907295 | 150 |
| 161 | 4 | 46784327 | 55802701 | 150 |
| 162 | 4 | 50930009 | 59585558 | 150 |
| 163 | 4 | 55834987 | 64643307 | 150 |
| 164 | 4 | 59756670 | 68754789 | 150 |
| 165 | 4 | 64660178 | 72771971 | 150 |
| 166 | 4 | 68802676 | 77421379 | 150 |
| 167 | 4 | 72874122 | 81978463 | 150 |
| 168 | 4 | 77591687 | 86406418 | 150 |
| 169 | 4 | 82021416 | 90424272 | 150 |
| 170 | 4 | 86445960 | 95640753 | 150 |
| 171 | 4 | 90498452 | 99577251 | 150 |
| 172 | 4 | 95835685 | 103605738 | 150 |
| 173 | 4 | 99626001 | 108036559 | 150 |
| 174 | 4 | 103671694 | 111559084 | 150 |
| 175 | 4 | 108076822 | 116726535 | 150 |
| 176 | 4 | 111571054 | 119253501 | 117 |
| 177 | 5 | 91775 | 10938908 | 150 |
| 178 | 5 | 4487167 | 16154097 | 150 |
| 179 | 5 | 10988952 | 21775869 | 150 |
| 180 | 5 | 16203101 | 26061543 | 150 |
| 181 | 5 | 21971548 | 30213508 | 150 |
| 182 | 5 | 26098980 | 34212680 | 150 |
| 183 | 5 | 30235737 | 40973401 | 150 |
| 184 | 5 | 34323465 | 45696296 | 150 |
| 185 | 5 | 41162117 | 50504101 | 150 |
| 186 | 5 | 45779648 | 55392813 | 150 |
| 187 | 5 | 50525574 | 60933309 | 150 |
| 188 | 5 | 55447086 | 65104723 | 150 |
| 189 | 5 | 60988809 | 69235412 | 150 |
| 190 | 5 | 65150264 | 73229489 | 150 |
| 191 | 5 | 69279532 | 77722326 | 150 |
| 192 | 5 | 73283220 | 82166580 | 150 |
| 193 | 5 | 77767268 | 86658080 | 150 |
| 194 | 5 | 82187017 | 90912960 | 150 |
| 195 | 5 | 86726320 | 95411245 | 150 |
| 196 | 5 | 90958314 | 99727022 | 150 |
| 197 | 5 | 95430614 | 103509118 | 150 |
| 198 | 5 | 99759486 | 106866039 | 127 |
| 199 | 6 | 313059 | 11044297 | 150 |
| 200 | 6 | 6775345 | 15205186 | 150 |
| 201 | 6 | 11075526 | 19179130 | 150 |
| 202 | 6 | 15411332 | 23248198 | 150 |
| 203 | 6 | 19333068 | 27978970 | 150 |
| 204 | 6 | 23277124 | 32594843 | 150 |
| 205 | 6 | 28123410 | 38906528 | 150 |
| 206 | 6 | 32615209 | 43798415 | 150 |
| 207 | 6 | 38952950 | 48762234 | 150 |
| 208 | 6 | 43818397 | 53485546 | 150 |
| 209 | 6 | 48825085 | 58421718 | 150 |
| 210 | 6 | 53826107 | 63013613 | 150 |
| 211 | 6 | 58445946 | 68565174 | 150 |
| 212 | 6 | 63053163 | 73869965 | 150 |
| 213 | 6 | 68760044 | 80148921 | 150 |
| 214 | 6 | 73950556 | 86242572 | 150 |
| 215 | 6 | 80186014 | 90861638 | 150 |
| 216 | 6 | 86268756 | 95475108 | 150 |
| 217 | 6 | 90972566 | 99698575 | 150 |
| 218 | 6 | 95526357 | 103607407 | 150 |
| 219 | 6 | 99805125 | 108291988 | 150 |
| 220 | 6 | 103648996 | 112817744 | 150 |
| 221 | 6 | 108376089 | 116668852 | 134 |
| 222 | 7 | 516431 | 8553422 | 150 |
| 223 | 7 | 4425742 | 12628497 | 150 |
| 224 | 7 | 8603908 | 17834128 | 150 |
| 225 | 7 | 12709123 | 22088616 | 150 |
| 226 | 7 | 17873014 | 26745110 | 150 |
| 227 | 7 | 22097462 | 31127673 | 150 |
| 228 | 7 | 26755264 | 35920538 | 150 |
| 229 | 7 | 31225687 | 40201773 | 150 |
| 230 | 7 | 35942157 | 45432575 | 150 |
| 231 | 7 | 40231558 | 49427992 | 150 |
| 232 | 7 | 45478746 | 53702036 | 150 |
| 233 | 7 | 49514996 | 58649028 | 150 |
| 234 | 7 | 53757165 | 63814165 | 150 |
| 235 | 7 | 58720023 | 67897432 | 150 |
| 236 | 7 | 64034963 | 72321943 | 150 |
| 237 | 7 | 67968976 | 76514110 | 150 |
| 238 | 7 | 72326425 | 81358932 | 150 |
| 239 | 7 | 76612681 | 85967358 | 150 |
| 240 | 7 | 81383811 | 89876831 | 150 |
| 241 | 7 | 85992070 | 94475616 | 150 |
| 242 | 7 | 89934677 | 99733325 | 150 |
| 243 | 8 | 2459 | 9150843 | 150 |
| 244 | 8 | 4742187 | 13698060 | 150 |
| 245 | 8 | 9175222 | 17014826 | 150 |
| 246 | 8 | 13734177 | 21237135 | 150 |
| 247 | 8 | 17108280 | 26186133 | 150 |
| 248 | 8 | 21344959 | 30656582 | 150 |
| 249 | 8 | 26292338 | 35299284 | 150 |
| 250 | 8 | 30712681 | 39284022 | 150 |
| 251 | 8 | 35355939 | 43831662 | 150 |
| 252 | 8 | 39344358 | 47913151 | 150 |
| 253 | 8 | 43866532 | 53493005 | 150 |
| 254 | 8 | 47947284 | 57996793 | 150 |
| 255 | 8 | 53564821 | 62037915 | 150 |
| 256 | 8 | 58031122 | 66676250 | 150 |
| 257 | 8 | 62102248 | 71224124 | 150 |
| 258 | 8 | 66754844 | 75847021 | 150 |
| 259 | 8 | 71264159 | 79990698 | 150 |
| 260 | 8 | 75906185 | 84205645 | 150 |
| 261 | 8 | 80021295 | 88291901 | 150 |
| 262 | 9 | 530741 | 9914782 | 150 |
| 263 | 9 | 5432369 | 15213397 | 150 |
| 264 | 9 | 9945328 | 19549746 | 150 |
| 265 | 9 | 15245885 | 23196104 | 150 |
| 266 | 9 | 19630364 | 27593399 | 150 |
| 267 | 9 | 23248081 | 31944220 | 150 |
| 268 | 9 | 27636855 | 35857204 | 150 |
| 269 | 9 | 32003055 | 40498168 | 150 |
| 270 | 9 | 36000391 | 45250113 | 150 |
| 271 | 9 | 40660629 | 52221945 | 150 |
| 272 | 9 | 45306636 | 56406931 | 150 |
| 273 | 9 | 52428789 | 60584570 | 150 |
| 274 | 9 | 56506969 | 65114468 | 150 |
| 275 | 9 | 60614133 | 69709423 | 150 |
| 276 | 9 | 65149940 | 74299897 | 150 |
| 277 | 9 | 69724659 | 78826196 | 150 |
| 278 | 9 | 74309305 | 82914168 | 150 |
| 279 | 9 | 78846991 | 87056637 | 150 |
| 280 | 9 | 82927581 | 91767918 | 150 |
| 281 | 9 | 87093273 | 94684385 | 121 |
| 282 | 10 | 1655720 | 11799294 | 150 |
| 283 | 10 | 6730970 | 16338178 | 150 |
| 284 | 10 | 11853718 | 20508567 | 150 |
| 285 | 10 | 16378011 | 24501714 | 150 |
| 286 | 10 | 20540068 | 28538940 | 150 |
| 287 | 10 | 24561101 | 32103055 | 150 |
| 288 | 10 | 28584727 | 36210255 | 150 |
| 289 | 10 | 32110323 | 41607668 | 150 |
| 290 | 10 | 36238012 | 46714199 | 150 |
| 291 | 10 | 41802553 | 50290499 | 150 |
| 292 | 10 | 46804626 | 54216568 | 150 |
| 293 | 10 | 50482176 | 58895153 | 150 |
| 294 | 10 | 54255353 | 63808989 | 150 |
| 295 | 10 | 58923133 | 68743197 | 150 |
| 296 | 10 | 63849945 | 74391164 | 150 |
| 297 | 10 | 68818754 | 78277655 | 150 |
| 298 | 10 | 74412897 | 82590071 | 150 |
| 299 | 10 | 78296997 | 86415081 | 141 |
| 300 | 11 | 679096 | 13474858 | 150 |
| 301 | 11 | 7626277 | 18248852 | 150 |
| 302 | 11 | 13506988 | 23088890 | 150 |
| 303 | 11 | 18255835 | 28779375 | 150 |
| 304 | 11 | 23383949 | 33722495 | 150 |
| 305 | 11 | 28846576 | 38656286 | 150 |
| 306 | 11 | 33733902 | 44221287 | 150 |
| 307 | 11 | 38743671 | 49277047 | 150 |
| 308 | 11 | 44231137 | 54556828 | 150 |
| 309 | 11 | 49332243 | 60239789 | 150 |
| 310 | 12 | 180295 | 11260159 | 150 |
| 311 | 12 | 6626081 | 15857081 | 150 |
| 312 | 12 | 11310194 | 20220769 | 150 |
| 313 | 12 | 15872744 | 25993613 | 150 |
| 314 | 12 | 20272553 | 29921374 | 150 |
| 315 | 12 | 26076376 | 34654736 | 150 |
| 316 | 12 | 30058029 | 40135863 | 150 |
| 317 | 12 | 34688821 | 44463092 | 150 |
| 318 | 12 | 40184013 | 48288842 | 150 |
| 319 | 12 | 44498233 | 54348772 | 150 |
| 320 | 12 | 48377650 | 59526277 | 150 |
| 321 | 12 | 54435941 | 64501140 | 150 |
| 322 | 12 | 59548483 | 68784914 | 150 |
| 323 | 12 | 64524300 | 72492559 | 150 |
| 324 | 12 | 68852951 | 77032530 | 150 |
| 325 | 13 | 183792 | 8920675 | 150 |
| 326 | 13 | 4712431 | 14396049 | 150 |
| 327 | 13 | 9182853 | 19450966 | 150 |
| 328 | 13 | 14534855 | 24339539 | 150 |
| 329 | 13 | 19491271 | 28553622 | 150 |
| 330 | 13 | 24346362 | 33301410 | 150 |
| 331 | 13 | 28595532 | 38630387 | 150 |
| 332 | 13 | 33580453 | 44193791 | 150 |
| 333 | 13 | 38741390 | 51028375 | 150 |
| 334 | 13 | 44268486 | 55660662 | 150 |
| 335 | 13 | 51044917 | 62269037 | 150 |
| 336 | 13 | 55748963 | 69621823 | 150 |
| 337 | 13 | 62335456 | 75194989 | 150 |
| 338 | 13 | 69666102 | 80611611 | 150 |
| 339 | 13 | 75367615 | 83038788 | 115 |
| 340 | 14 | 178239 | 12015494 | 150 |
| 341 | 14 | 5325961 | 17787859 | 150 |
| 342 | 14 | 12136051 | 23098569 | 150 |
| 343 | 14 | 17824183 | 28147316 | 150 |
| 344 | 14 | 23149102 | 33822083 | 150 |
| 345 | 14 | 28204607 | 39795104 | 150 |
| 346 | 14 | 33954641 | 45544769 | 150 |
| 347 | 14 | 39826395 | 52617343 | 150 |
| 348 | 14 | 45595860 | 59461660 | 150 |
| 349 | 15 | 790390 | 12465665 | 150 |
| 350 | 15 | 7078006 | 19135700 | 150 |
| 351 | 15 | 12505477 | 23816985 | 150 |
| 352 | 15 | 19205738 | 27795640 | 150 |
| 353 | 15 | 23845949 | 31857853 | 150 |
| 354 | 15 | 27808964 | 36615549 | 150 |
| 355 | 15 | 31874821 | 41001941 | 150 |
| 356 | 15 | 36648070 | 46116205 | 150 |
| 357 | 15 | 41030487 | 52571399 | 150 |
| 358 | 15 | 46133776 | 57440615 | 150 |
| 359 | 15 | 52602119 | 61846859 | 150 |
| 360 | 15 | 57555000 | 66440686 | 150 |
| 361 | 15 | 61910472 | 71115834 | 150 |
| 362 | 15 | 66469876 | 75842476 | 150 |
| 363 | 15 | 71180414 | 80435433 | 150 |
| 364 | 16 | 156698 | 9531484 | 150 |
| 365 | 16 | 5310236 | 15514961 | 150 |
| 366 | 16 | 9567145 | 20124774 | 150 |
| 367 | 16 | 15557272 | 25073161 | 150 |
| 368 | 16 | 20166035 | 29415369 | 150 |
| 369 | 16 | 25123129 | 34475296 | 150 |
| 370 | 16 | 29456587 | 39057236 | 150 |
| 371 | 16 | 34529062 | 43273047 | 150 |
| 372 | 16 | 39077896 | 48407318 | 150 |
| 373 | 16 | 43340338 | 54052490 | 150 |
| 374 | 16 | 48523137 | 58809785 | 150 |
| 375 | 16 | 54092131 | 64022502 | 150 |
| 376 | 16 | 58925398 | 68391888 | 150 |
| 377 | 16 | 64064879 | 71555691 | 116 |
| 378 | 17 | 47789 | 9840497 | 150 |
| 379 | 17 | 5569349 | 15084074 | 150 |
| 380 | 17 | 9855505 | 19837353 | 150 |
| 381 | 17 | 15145489 | 25713704 | 150 |
| 382 | 17 | 19982656 | 31448756 | 150 |
| 383 | 17 | 25826660 | 37452349 | 150 |
| 384 | 17 | 31498699 | 42131038 | 150 |
| 385 | 17 | 37485924 | 47313862 | 150 |
| 386 | 17 | 42191784 | 52092174 | 150 |
| 387 | 17 | 47416116 | 56753909 | 150 |
| 388 | 17 | 52130027 | 62152083 | 150 |
| 389 | 17 | 56786527 | 67905066 | 150 |
| 390 | 17 | 62257956 | 72121029 | 132 |
| 391 | 18 | 1129227 | 11701788 | 150 |
| 392 | 18 | 7064050 | 16264073 | 150 |
| 393 | 18 | 11743896 | 20991155 | 150 |
| 394 | 18 | 16359451 | 26037876 | 150 |
| 395 | 18 | 21136208 | 30839852 | 150 |
| 396 | 18 | 26093817 | 35993211 | 150 |
| 397 | 18 | 30892354 | 40617543 | 150 |
| 398 | 18 | 36045091 | 46100361 | 150 |
| 399 | 18 | 40678549 | 50931502 | 150 |
| 400 | 18 | 46138370 | 56180341 | 150 |
| 401 | 18 | 50970742 | 60853888 | 150 |
| 402 | 18 | 56212070 | 65625925 | 150 |
| 403 | 19 | 271033 | 10171209 | 150 |
| 404 | 19 | 5622653 | 15626135 | 150 |
| 405 | 19 | 10252706 | 21486164 | 150 |
| 406 | 19 | 15659795 | 27100926 | 150 |
| 407 | 19 | 21542256 | 32213913 | 150 |
| 408 | 19 | 27130032 | 36563885 | 150 |
| 409 | 19 | 32290813 | 41709054 | 150 |
| 410 | 19 | 36615520 | 46882636 | 150 |
| 411 | 19 | 41742622 | 53596723 | 150 |
| 412 | 19 | 46920272 | 58334807 | 150 |
| 413 | 20 | 216960 | 10294757 | 150 |
| 414 | 20 | 5661846 | 14620561 | 150 |
| 415 | 20 | 10362856 | 19988514 | 150 |
| 416 | 20 | 14663104 | 24145435 | 150 |
| 417 | 20 | 20027429 | 30410154 | 150 |
| 418 | 20 | 24176589 | 35239828 | 150 |
| 419 | 20 | 30501109 | 38937734 | 150 |
| 420 | 20 | 35251902 | 43116934 | 150 |
| 421 | 20 | 39053345 | 46958488 | 150 |
| 422 | 20 | 43161083 | 50943810 | 126 |
| 423 | 21 | 231657 | 11143699 | 150 |
| 424 | 21 | 6751357 | 15836532 | 150 |
| 425 | 21 | 11238464 | 22213120 | 150 |
| 426 | 21 | 15990368 | 28638407 | 150 |
| 427 | 21 | 22278372 | 34648689 | 150 |
| 428 | 21 | 28853040 | 43818339 | 150 |
| 429 | 21 | 34678893 | 50029788 | 137 |
| 430 | 22 | 259746 | 9669140 | 150 |
| 431 | 22 | 5808469 | 15045102 | 150 |
| 432 | 22 | 9725549 | 20081370 | 150 |
| 433 | 22 | 15070056 | 24605942 | 150 |
| 434 | 22 | 20110815 | 28082930 | 150 |
| 435 | 22 | 24643421 | 32963011 | 150 |
| 436 | 22 | 28171389 | 37710273 | 150 |
| 437 | 22 | 33014199 | 41919118 | 150 |
| 438 | 22 | 37729842 | 47408363 | 150 |
| 439 | 22 | 41984735 | 50696336 | 129 |
| 440 | 23 | 306898 | 11303094 | 150 |
| 441 | 23 | 5823835 | 18138292 | 150 |
| 442 | 23 | 11343844 | 24092259 | 150 |
| 443 | 23 | 18181618 | 31600741 | 150 |
| 444 | 23 | 24204428 | 37717812 | 150 |
| 445 | 23 | 31778945 | 44559717 | 150 |
| 446 | 23 | 37885994 | 50666507 | 150 |
| 447 | 23 | 44696146 | 56674506 | 150 |
| 448 | 23 | 50695482 | 62214575 | 141 |
| 449 | 24 | 96467 | 14785896 | 150 |
| 450 | 24 | 6558653 | 22021018 | 150 |
| 451 | 24 | 14838850 | 29007948 | 150 |
| 452 | 24 | 22377381 | 35585230 | 150 |
| 453 | 24 | 29098444 | 42027686 | 139 |
| 454 | 25 | 79217 | 10602294 | 150 |
| 455 | 25 | 6143734 | 15471078 | 150 |
| 456 | 25 | 10627436 | 20664988 | 150 |
| 457 | 25 | 15527672 | 25988024 | 150 |
| 458 | 25 | 20692190 | 31037322 | 150 |
| 459 | 25 | 26031277 | 35354809 | 150 |
| 460 | 25 | 31066943 | 40184179 | 150 |
| 461 | 25 | 35403409 | 44534000 | 150 |
| 462 | 26 | 166516 | 10854951 | 150 |
| 463 | 26 | 5426933 | 16390436 | 150 |
| 464 | 26 | 10883077 | 22543425 | 150 |
| 465 | 26 | 16448375 | 27177792 | 150 |
| 466 | 26 | 22586410 | 31875529 | 150 |
| 467 | 26 | 27216096 | 37050856 | 150 |
| 468 | 26 | 31939957 | 42311012 | 150 |

**Table S3:** List of domestic sheep breeds used in the haplotype-sharing analyses

| **Sheep Breed** | **Abbreviation** | **Number of sheep** |
| --- | --- | --- |
| African Dorper | ADP | 21 |
| African White Dorper | AWD | 6 |
| Afshari | AFS | 37 |
| Altamurana | ALT | 24 |
| Australian Coopworth | CPW | 19 |
| Australian Industry Merino | MER | 88 |
| Australian Merino | MER | 50 |
| Australian Poll Dorset | APD | 108 |
| Australian Poll Merino | APM | 98 |
| Australian Suffolk | ASU | 109 |
| Bangladeshi BGE | BGE | 24 |
| Bangladeshi Garole | BGA | 24 |
| Barbados BlackBelly | BBB | 24 |
| BlackHeaded Mountain | BHM | 24 |
| Border Leicester | BRL | 48 |
| Boreray | BOR | 17 |
| Brazilian Creole | BCS | 23 |
| Bundneroberlander Sheep | BOS | 24 |
| Castellana | CAS | 23 |
| Changthangi | CHA | 29 |
| Chinese Merino | CME | 23 |
| Chios | CHI | 23 |
| Churra | CHU | 120 |
| Comisana | COM | 24 |
| Cyprus Fat Tail | CFT | 30 |
| Deccani | IDC | 24 |
| DorsetHorn | DSH | 21 |
| East Friesian Brown | EFB | 39 |
| East Friesian White | EFW | 9 |
| Engadine Red Sheep | ERS | 24 |
| Ethiopian Menz | EMZ | 34 |
| Finnsheep | FIN | 99 |
| Galway | GAL | 49 |
| Garut | GUR | 22 |
| German Texel | GTX | 46 |
| Gulf Coast Native | GCN | 94 |
| Indian Garole | GAR | 26 |
| Irish Suffolk | ISF | 55 |
| Karakas | KRS | 18 |
| Leccese | LEC | 24 |
| Macarthur Merino | MCM | 10 |
| Meat Lacaune | LAC | 78 |
| Merinolandschaf | MLA | 24 |
| Milk Lacaune | LAC | 103 |
| Moghani | MOG | 34 |
| Morada Nova | BMN | 22 |
| Namaqua Afrikaner | NQA | 12 |
| New Zealand Romney | ROM | 24 |
| New Zealand Texel | NTX | 24 |
| Norduz | NDZ | 20 |
| Ojalada | OJA | 24 |
| Old Norwegian spaelsau | NSP | 15 |
| Qezel | QEZ | 35 |
| Rambouillet | RMB | 102 |
| Rasaaragonesa | RAA | 22 |
| Red Maasai | RMA | 45 |
| Ronderib Afrikaner | RDA | 17 |
| Sakiz | SKZ | 22 |
| Santa Ines | BSI | 47 |
| Sardinian Ancestral Black | SAB | 20 |
| Scottish Blackface | SBF | 56 |
| Scottish Texel | STX | 80 |
| Spael-coloured | NSP | 3 |
| Spael-white | NSP | 32 |
| St Elizabeth | STE | 10 |
| Sumatra | SUM | 24 |
| Swiss Black-Brown Mountain Sheep | SBS | 24 |
| Swiss Mirror Sheep | SMS | 24 |
| Swiss White Alpine Sheep | SWA | 24 |
| Tibetan | TIB | 37 |
| Valais Blacknose Sheep | VBS | 24 |
| Valais Red Sheep | VRS | 24 |
| Wiltshire | WIL | 23 |

**Table S4:** List of the 15 SNPs showing strongest associations using GWAS for each trait. Results were obtained using the *qtscore* function in the R package GenABEL (Aulchenko *et al* 2007). The additive effect of the minor are in mm, except for weight, for which the additive effect is in kg.

| **Trait** | **SNP** | **Chromosome** | **Position** | **Major allele** | **Minor allele** | **Number of observations** | **Additive effect of the minor allele** | **S** | **Χ^2^** | **P** |
| --- | --- | --- | --- | --- | --- | --- | --- | --- | --- | --- |
| Foreleg | s63944.1 | 16 | 69135141 | A | G | 887 | -0.676 | 0.143 | 22.262 | 2.38 x 10-06 |
|  | s23172.1 | 16 | 69726554 | A | G | 885 | -0.673 | 0.144 | 21.788 | 3.04 x 10-06 |
|  | s22142.1 | 16 | 69679810 | G | A | 887 | -0.657 | 0.143 | 21.223 | 4.09 x 10-06 |
|  | s74894.1 | 19 | 52470202 | G | A | 886 | -0.405 | 0.097 | 17.352 | 3.11 x 10-05 |
|  | s70233.1 | 16 | 66776169 | G | A | 883 | -0.95 | 0.234 | 16.528 | 4.79 x 10-05 |
|  | OAR16_74353353.1 | 16 | 68454044 | A | G | 887 | -0.716 | 0.179 | 15.95 | 6.50 x 10-05 |
|  | s48811.1 | 7 | 84579439 | G | A | 880 | -0.351 | 0.089 | 15.409 | 8.66 x 10-05 |
|  | s20693.1 | 16 | 69657258 | A | G | 873 | -0.357 | 0.092 | 14.936 | 1.11 x 10-04 |
|  | s50107.1 | 9 | 50463148 | A | G | 887 | -0.225 | 0.06 | 14.298 | 1.56 x 10-04 |
|  | s49039.1 | 9 | 50472159 | A | G | 887 | -0.214 | 0.06 | 12.815 | 3.44 x 10-04 |
|  | OAR9_54274940.1 | 9 | 51885382 | A | C | 886 | 0.209 | 0.062 | 11.508 | 6.93 x 10-04 |
|  | s13613.1 | 1 | 37304186 | G | A | 886 | 0.429 | 0.129 | 11.135 | 8.47 x 10-04 |
|  | s53565.1 | 16 | 70710282 | A | G | 886 | -0.36 | 0.108 | 11.023 | 9.00 x 10-04 |
|  | OAR2_81544695.1 | 2 | 76545527 | G | A | 887 | 0.198 | 0.061 | 10.463 | 1.22 x 10-03 |
|  | OAR20_41820631.1 | 20 | 38355175 | A | G | 887 | -0.282 | 0.087 | 10.455 | 1.22 x 10-03 |
| Hindleg | s23172.1 | 16 | 69726554 | A | G | 897 | -0.445 | 0.084 | 28.224 | **1.08 x 10-07** |
|  | s22142.1 | 16 | 69679810 | G | A | 899 | -0.432 | 0.083 | 27.274 | **1.77 x 10-07** |
|  | s63944.1 | 16 | 69135141 | A | G | 899 | -0.431 | 0.083 | 26.824 | **2.23 x 10-07** |
|  | OAR16_74353353.1 | 16 | 68454044 | A | G | 899 | -0.471 | 0.104 | 20.454 | 6.11 x 10-06 |
|  | s70233.1 | 16 | 66776169 | G | A | 895 | -0.588 | 0.136 | 18.557 | 1.65 x 10-05 |
|  | s53565.1 | 16 | 70710282 | A | G | 898 | -0.268 | 0.063 | 18.108 | 2.09 x 10-05 |
|  | s20693.1 | 16 | 69657258 | A | G | 885 | -0.221 | 0.054 | 16.933 | 3.87 x 10-05 |
|  | OAR16_75450521.1 | 16 | 69490404 | G | A | 899 | -0.242 | 0.059 | 16.551 | 4.73 x 10-05 |
|  | OAR6_80909611.1 | 6 | 74098684 | A | C | 898 | 0.132 | 0.035 | 14.482 | 1.41 x 10-04 |
|  | s75789.1 | 16 | 67852018 | G | A | 899 | -0.469 | 0.125 | 13.988 | 1.84 x 10-04 |
|  | s15712.1 | 16 | 69408377 | G | A | 899 | -0.16 | 0.043 | 13.643 | 2.21 x 10-04 |
|  | s74894.1 | 19 | 52470202 | G | A | 898 | -0.201 | 0.056 | 12.792 | 3.48 x 10-04 |
|  | OAR20_39945404.1 | 20 | 36501574 | G | A | 899 | -0.2 | 0.058 | 11.733 | 6.14 x 10-04 |
|  | OAR8_21072066.1 | 8 | 18757172 | G | A | 898 | 0.118 | 0.035 | 11.044 | 8.90 x 10-04 |
|  | s15898.1 | 7 | 56115836 | G | A | 899 | 0.145 | 0.045 | 10.387 | 1.27 x 10-03 |
| Metacarpal | s23172.1 | 16 | 69726554 | A | G | 937 | -1.476 | 0.233 | 40.079 | **2.44 x 10-10** |
|  | s22142.1 | 16 | 69679810 | G | A | 940 | -1.45 | 0.23 | 39.809 | **2.80 x 10-10** |
|  | s63944.1 | 16 | 69135141 | A | G | 940 | -1.366 | 0.23 | 35.336 | **2.77 x 10-09** |
|  | s74894.1 | 19 | 52470202 | G | A | 939 | -0.966 | 0.173 | 31.195 | **2.33 x 10-08** |
|  | OAR16_74353353.1 | 16 | 68454044 | A | G | 940 | -1.321 | 0.277 | 22.716 | 1.88 x 10-06 |
|  | OAR16_75450521.1 | 16 | 69490404 | G | A | 940 | -0.741 | 0.167 | 19.637 | 9.37 x 10-06 |
|  | OAR19_57613919.1 | 19 | 54408551 | A | C | 926 | -0.616 | 0.158 | 15.28 | 9.27 x 10-05 |
|  | OAR19_57094840.1 | 19 | 53903165 | A | C | 938 | -0.6 | 0.155 | 15.032 | 1.06 x 10-04 |
|  | s53565.1 | 16 | 70710282 | A | G | 937 | -0.673 | 0.176 | 14.604 | 1.33 x 10-04 |
|  | s75789.1 | 16 | 67852018 | G | A | 940 | -1.178 | 0.311 | 14.328 | 1.54 x 10-04 |
|  | OAR19_57822817.1 | 19 | 54580633 | A | G | 940 | -0.566 | 0.154 | 13.564 | 2.31 x 10-04 |
|  | s31207.1 | 19 | 54331442 | G | A | 939 | -0.566 | 0.154 | 13.517 | 2.36 x 10-04 |
|  | OAR19_57425358.1 | 19 | 54231255 | G | A | 940 | -0.562 | 0.154 | 13.305 | 2.65 x 10-04 |
|  | s20693.1 | 16 | 69657258 | A | G | 929 | -0.532 | 0.148 | 12.854 | 3.37 x 10-04 |
|  | s41950.1 | 19 | 49391499 | G | A | 940 | -0.524 | 0.147 | 12.713 | 3.63 x 10-04 |
| Weight | s25262.1 | 3 | 78405637 | A | G | 896 | 0.125 | 0.035 | 13.013 | 3.09 x 10-04 |
|  | OAR26_27050858.1 | 26 | 23103698 | A | G | 897 | -0.118 | 0.033 | 12.985 | 3.14 x 10-04 |
|  | OAR3_82856188.1 | 3 | 78326240 | A | G | 897 | 0.123 | 0.034 | 12.911 | 3.27 x 10-04 |
|  | s21478.1 | 3 | 78369921 | A | G | 896 | 0.124 | 0.034 | 12.876 | 3.33 x 10-04 |
|  | OAR6_22324973.1 | 6 | 19333068 | A | G | 897 | 0.105 | 0.029 | 12.603 | 3.85 x 10-04 |
|  | OAR6_21480732.1 | 6 | 18513309 | G | A | 897 | 0.109 | 0.031 | 12.573 | 3.91 x 10-04 |
|  | DU209581_415.1 | 6 | 18596986 | G | C | 897 | 0.109 | 0.031 | 12.573 | 3.91 x 10-04 |
|  | OAR6_21621054.1 | 6 | 18666919 | G | A | 897 | 0.109 | 0.031 | 12.573 | 3.91 x 10-04 |
|  | OAR26_31479674.1 | 26 | 27363890 | A | G | 896 | 0.096 | 0.027 | 12.562 | 3.94 x 10-04 |
|  | OAR6_21943640.1 | 6 | 18982682 | G | A | 897 | 0.109 | 0.031 | 12.492 | 4.09 x 10-04 |
|  | s70780.1 | 6 | 18414353 | G | A | 896 | 0.108 | 0.031 | 12.32 | 4.48 x 10-04 |
|  | OAR6_21422656.1 | 6 | 18447640 | G | A | 897 | 0.103 | 0.03 | 11.784 | 5.97 x 10-04 |
|  | OAR7_36652030.1 | 7 | 32477867 | G | A | 897 | 0.114 | 0.033 | 11.686 | 6.30 x 10-04 |
|  | OAR6_53042815.1 | 6 | 48091453 | G | A | 897 | -0.182 | 0.054 | 11.495 | 6.98 x 10-04 |
|  | OAR10_2742625.1 | 10 | 4903158 | G | A | 897 | -0.099 | 0.029 | 11.406 | 7.32 x 10-04 |
| Jaw | OAR10_24438051.1 | 10 | 24582181 | C | A | 1020 | 1.132 | 0.35 | 10.457 | 1.22 x 10-03 |
|  | OAR10_24891503.1 | 10 | 24977576 | A | G | 1020 | 1.132 | 0.35 | 10.457 | 1.22 x 10-03 |
|  | OAR16_41737441.1 | 16 | 38473232 | A | C | 1012 | -0.877 | 0.278 | 9.962 | 1.60 x 10-03 |
|  | s61513.1 | 5 | 1510848 | C | A | 1020 | 0.297 | 0.095 | 9.766 | 1.78 x 10-03 |
|  | s40078.1 | 15 | 36615549 | A | G | 1014 | -1.258 | 0.404 | 9.699 | 1.84 x 10-03 |
|  | s33893.1 | 1 | 38960363 | G | A | 1020 | -0.453 | 0.146 | 9.589 | 1.96 x 10-03 |
|  | OAR20_50429719.1 | 20 | 46391556 | A | C | 1020 | 0.298 | 0.099 | 9.083 | 2.58 x 10-03 |
|  | OAR1_212938417.1 | 1 | 1.97E+08 | A | C | 1020 | -1.026 | 0.342 | 9.019 | 2.67 x 10-03 |
|  | OAR15_27908490.1 | 15 | 26593448 | A | C | 1019 | -0.563 | 0.193 | 8.475 | 3.60 x 10-03 |
|  | OAR14_63480723.1 | 14 | 58176852 | A | G | 1020 | -0.399 | 0.138 | 8.423 | 3.70 x 10-03 |
|  | OAR11_27165440.1 | 11 | 25984725 | G | A | 1019 | 0.425 | 0.147 | 8.311 | 3.94 x 10-03 |
|  | OAR4_42883086.1 | 4 | 40781234 | A | G | 1020 | -0.913 | 0.32 | 8.128 | 4.36 x 10-03 |
|  | OAR14_4353963.1 | 14 | 4422141 | A | C | 1018 | 0.301 | 0.105 | 8.115 | 4.39 x 10-03 |
|  | OAR6_38840563.1 | 6 | 34712230 | G | A | 1020 | 0.291 | 0.102 | 8.075 | 4.49 x 10-03 |
|  | OAR6_38863569.1 | 6 | 34744791 | A | C | 1017 | 0.291 | 0.102 | 8.068 | 4.50 x 10-03 |

**Table S5:** Parameter estimates for fixed effects from sex-specific Bayesian models of annual survival, number of annual recruits, lifetime breeding success (LBS) and lifetime number of recruits (LR). Fixed effect contrasts are relative to the model intercept, which represents the major homozygote genotypes (AA and GG for s23172.1 and s74894.1 respectively) conferring longer legs. Significant terms are highlighted in bold. Sample sizes for each fitness measure are shown, and give the total number of individuals, and the total number of observations for each SNP between parentheses. The numbers show the major homozygotes, heterozygotes and minor homozygotes respectively.

| Fitness measure | Fixed effect term | SNP genotype | SNP sample sizes | MCMC estimates | pMCMC |
| --- | --- | --- | --- | --- | --- |
| Male survival | (Intercept) |  |  | -1.708 (-5.096-1.583) | 0.288 |
|  | Maternal age |  |  | 1.014 (0.159-1.918) | **0.018** |
|  | Maternal age (quadratic term) |  |  | -0.093 (-0.175--0.024) | **0.01** |
|  | Litter size |  |  | -2.587 (-4.15--1.055) | **0.001** |
|  | Age |  |  | 0.769 (-0.183-1.709) | 0.148 |
|  | Age (quadratic term) |  |  | -0.221 (-0.291--0.152) | **0.001** |
|  | s23172.1 | AG | 1105/94/4 (2336/168/8) | -1.145 (-3.066-0.551) | 0.208 |
|  | s23172.1 | GG |  | -1.927 (-11.503-6.024) | 0.666 |
|  | s74894.1 | GA | 969/224/10 (2022/466/24) | 0.328 (-0.815-1.503) | 0.58 |
|  | s74894.1 | AA |  | -0.52 (-4.981-4.709) | 0.816 |
| Female survival | (Intercept) |  |  | -3.77 (-7.739--0.688) | **0.032** |
|  | Maternal age |  |  | 1.819 (0.759-3.013) | **0.001** |
|  | Maternal age (quadratic term) |  |  | -0.131 (-0.221--0.038) | **0.001** |
|  | Litter size |  |  | -4.909 (-7.055--2.884) | **0.001** |
|  | Age |  |  | 0.937 (0.529-1.326) | **0.001** |
|  | Age (quadratic term) |  |  | -0.216 (-0.256--0.169) | **0.001** |
|  | s23172.1 | AG | 1314/102/2 (4469/354/9) | 0.48 (-2.002-2.672) | 0.678 |
|  | s23172.1 | GG |  | -1.256 (-15.595-10.481) | 0.87 |
|  | s74894.1 | GA | 1162/242/14 (3867/928/37) | 1.206 (-0.44-2.791) | 0.13 |
|  | s74894.1 | AA |  | -2.391 (-8.864-3.723) | 0.452 |
| Male recruits | (Intercept) |  |  | -4.309 (-4.997--3.71) | **0.001** |
|  | Maternal age |  |  | 0.291 (0.086-0.505) | **0.01** |
|  | Maternal age (quadratic term) |  |  | -0.027 (-0.045--0.008) | **0.004** |
|  | Litter size |  |  | -0.357 (-0.654--0.066) | **0.02** |
|  | Age |  |  | 1.127 (1.005-1.24) | **0.001** |
|  | Age (quadratic term) |  |  | -0.076 (-0.087--0.063) | **0.001** |
|  | s23172.1 | AG | 1105/94/4 (2336/168/8) | 0.17 (-0.242-0.594) | 0.426 |
|  | s23172.1 | GG |  | -0.606 (-2.911-1.21) | 0.564 |
|  | s74894.1 | GA | 969/224/10 (2022/466/24) | 0.089 (-0.213-0.359) | 0.512 |
|  | s74894.1 | AA |  | -0.148 (-1.294-0.989) | 0.812 |
| Female recruits | (Intercept) |  |  | -1.951 (-2.197--1.703) | **0.001** |
|  | Maternal age |  |  | 0.081 (0.017-0.152) | **0.026** |
|  | Maternal age (quadratic term) |  |  | -0.006 (-0.011-0) | **0.034** |
|  | Litter size |  |  | -0.123 (-0.234--0.014) | **0.028** |
|  | Age |  |  | 0.572 (0.515-0.621) | **0.001** |
|  | Age (quadratic term) |  |  | -0.049 (-0.053--0.043) | **0.001** |
|  | s23172.1 | AG | 1314/102/2 (4469/354/9) | 0.009 (-0.137-0.142) | 0.878 |
|  | s23172.1 | GG |  | 0.35 (-0.406-1.047) | 0.362 |
|  | s74894.1 | GA | 1162/242/14 (3867/928/37) | 0.049 (-0.035-0.148) | 0.314 |
|  | s74894.1 | AA |  | 0.154 (-0.319-0.557) | 0.506 |
| Male LBS | (Intercept) |  |  | -5.501 (-7.436--3.881) | **0.001** |
|  | Maternal age |  |  | 1.176 (0.716-1.663) | **0.001** |
|  | Maternal age (quadratic term) |  |  | -0.099 (-0.14--0.054) | **0.001** |
|  | Litter size |  |  | -1.104 (-1.798--0.506) | **0.002** |
|  | s23172.1 | AG | 919/92/2 | -0.303 (-1.271-0.583) | 0.526 |
|  | s23172.1 | GG |  | 1.97 (-2.32-6.168) | 0.364 |
|  | s74894.1 | GA | 837/170/6 | 0.206 (-0.399-0.757) | 0.526 |
|  | s74894.1 | AA |  | -1.925 (-5.911-1.831) | 0.298 |
| Female LBS | (Intercept) |  |  | -3.09 (-4.173--2.093) | **0.001** |
|  | Maternal age |  |  | 0.906 (0.682-1.179) | **0.001** |
|  | Maternal age (quadratic term) |  |  | -0.065 (-0.088--0.047) | **0.001** |
|  | Litter size |  |  | -1.201 (-1.644--0.806) | **0.001** |
|  | s23172.1 | AG | 986/86/3 | -0.078 (-0.624-0.54) | 0.82 |
|  | s23172.1 | GG |  | 2.29 (-0.182-4.617) | 0.066 |
|  | s74894.1 | GA | 887/179/9 | 0.494 (0.093-0.884) | **0.008** |
|  | s74894.1 | AA |  | -0.576 (-2.223-1.207) | 0.546 |
| Male LR | (Intercept) |  |  | -3.314 (-4.931--1.363) | **0.001** |
|  | Maternal age |  |  | 0.502 (0.005-0.999) | **0.042** |
|  | Maternal age (quadratic term) |  |  | -0.044 (-0.087-0) | 0.05 |
|  | Litter size |  |  | -0.817 (-1.411--0.243) | **0.014** |
|  | s23172.1 | AG | 734/66/2 | -0.472 (-1.37-0.587) | 0.342 |
|  | s23172.1 | GG |  | 1.328 (-2.665-5.245) | 0.482 |
|  | s74894.1 | GA | 659/138/5 | 0.296 (-0.28-0.969) | 0.332 |
|  | s74894.1 | AA |  | -2.14 (-5.908-1.599) | 0.26 |
| Female LR | (Intercept) |  |  | -1.479 (-2.417--0.443) | **0.006** |
|  | Maternal age |  |  | 0.44 (0.19-0.649) | **0.002** |
|  | Maternal age (quadratic term) |  |  | -0.03 (-0.048--0.01) | **0.004** |
|  | Litter size |  |  | -0.911 (-1.267--0.531) | **0.001** |
|  | s23172.1 | AG | 800/67/2 | 0.021 (-0.496-0.568) | 0.928 |
|  | s23172.1 | GG |  | 0.527 (-2.062-2.81) | 0.68 |
|  | s74894.1 | GA | 720/141/8 | 0.622 (0.265-1.018) | **0.001** |
|  | s74894.1 | AA |  | -0.327 (-1.913-1.004) | 0.686 |

**Table S6:** Selection coefficients for sex-specific annual survival, annual recruitment, lifetime breeding success (LBS) and lifetime number of recruits (LR) at genotypes at two SNPs which were genome-wide significant for leg length.

| **Fitness measure** | **SNP** | **Genotype^a^** | **Mean fitness** | **Relative fitness** | **Selection coefficient^b^** | **Sample size^c^** | **Equilibrium frequency^b^** |
| --- | --- | --- | --- | --- | --- | --- | --- |
| Male survival | s23172.1 | AA | 0.634 | 1 | 0 (0-0.371) | 1370 (2857) |  |
|  |  | AG | 0.544 | 0.858 | 0.142 (0-0.373) | 127 (226) |  |
|  |  | GG | 0.5 | 0.788 | 0.212 (0-1) | 4 (8) |  |
|  | s74894.1 | GG | 0.625 | 0.978 | 0.022 (0-0.224) | 1223 (2509) |  |
|  |  | GA | 0.639 | 1 | 0 (0-0.231) | 269 (562) | 0.38 (0-1) |
|  |  | AA | 0.615 | 0.963 | 0.037 (0-0.551) | 12 (26) |  |
| Female survival | s23172.1 | AA | 0.772 | 1 | 0 (0-0.115) | 1513 (5247) |  |
|  |  | AG | 0.769 | 0.995 | 0.005 (0-0.103) | 118 (415) |  |
|  |  | GG | 0.7 | 0.906 | 0.094 (0-0.321) | 3 (10) |  |
|  | s74894.1 | GG | 0.763 | 0.984 | 0.016 (0-0.037) | 1392 (4614) |  |
|  |  | GA | 0.775 | 1 | 0 (0-0.039) | 314 (1108) | 0.15(0-1) |
|  |  | AA | 0.702 | 0.906 | 0.094 (0-0.048) | 17 (47) |  |
| Male recruitment | s23172.1 | AA | 0.363 | 1 | 0 (0-0.755) | 1370 (2857) |  |
|  |  | AG | 0.301 | 0.829 | 0.171 (0-0.778) | 127 (226) |  |
|  |  | GG | 0.188 | 0.517 | 0.483 (0-1) | 4 (8) |  |
|  | s74894.1 | GG | 0.348 | 0.85 | 0.15 (0-0.705) | 1223 (2509) |  |
|  |  | GA | 0.409 | 1 | 0 (0-0.7) | 269 (562) | 0.19 (0-1) |
|  |  | AA | 0.154 | 0.376 | 0.624 (0-1) | 12 (26) |  |
| Female recruitment | s23172.1 | AA | 0.307 | 0.682 | 0.318 (0-0.307) | 1513 (5247) |  |
|  |  | AG | 0.3 | 0.667 | 0.333 (0-0.307) | 118 (415) |  |
|  |  | GG | 0.45 | 1 | 0 (0-1) | 3 (10) |  |
|  | s74894.1 | GG | 0.299 | 0.938 | 0.062 (0-0.147) | 1392 (4614) |  |
|  |  | GA | 0.315 | 0.986 | 0.014 (0-0.153) | 314 (1108) |  |
|  |  | AA | 0.319 | 1 | 0 (0-0.215) | 17 (47) |  |
| Male LBS | s23172.1 | AA | 1.992 | 1 | 0 (0-0.863) | 1283 |  |
|  |  | AG | 1.591 | 0.799 | 0.201 (0-0.878) | 132 |  |
|  |  | GG | 1.5 | 0.753 | 0.247 (0-1) | 2 |  |
|  | s74894.1 | GG | 1.905 | 0.849 | 0.151 (0-0.76) | 1174 |  |
|  |  | GA | 2.243 | 1 | 0 (0-0.77) | 235 | 0.18 (0-1) |
|  |  | AA | 0.75 | 0.334 | 0.666 (0-1) | 8 |  |
| Female LBS | s23172.1 | AA | 2.438 | 0.43 | 0.57 (0-0.679) | 1153 |  |
|  |  | AG | 2.02 | 0.357 | 0.643 (0-0.698) | 99 |  |
|  |  | GG | 5.667 | 1 | 0 (0-1) | 3 |  |
|  | s74894.1 | GG | 2.339 | 0.847 | 0.153 (0-0.53) | 1027 |  |
|  |  | GA | 2.761 | 1 | 0 (0-0.559) | 218 | 0.54 (0-1) |
|  |  | AA | 2.4 | 0.869 | 0.131 (0-0.805) | 10 |  |
| Male LR | s23172.1 | AA | 1.911 | 1 | 0 (0-0.884) | 921 |  |
|  |  | AG | 1.303 | 0.682 | 0.318 (0-0.91) | 89 |  |
|  |  | GG | 1.5 | 0.785 | 0.215 (0-1) | 2 |  |
|  | s74894.1 | GG | 1.753 | 0.716 | 0.284 (0-0.806) | 837 |  |
|  |  | GA | 2.446 | 1 | 0 (0-0.826) | 168 | 0.23 (0-1) |
|  |  | AA | 0.143 | 0.058 | 0.942 (0-1) | 7 |  |
| Female LR | s23172.1 | AA | 2.791 | 0.62 | 0.38 (0-0.702) | 928 |  |
|  |  | AG | 2.493 | 0.554 | 0.446 (0-0.709) | 75 |  |
|  |  | GG | 4.5 | 1 | 0 (0-1) | 2 |  |
|  | s74894.1 | GG | 2.66 | 0.803 | 0.197 (0-0.49) | 824 |  |
|  |  |  | 3.314 | 1 | 0 (0-0.494) | 172 | 0.50 (0-1) |
|  |  |  | 2.667 | 0.805 | 0.195 (0-0.805) | 9 |  |

^a^ For each SNP genotypes are ordered such that the first genotype is the homozygote genotype for the major alleles conferring longer legs.

^b^ 95% bootstrap confidence intervals are shown between parentheses.

^c^ The number of individuals in each SNP category. For fitness measures with repeated measures per individual, the total number of observations is shown between parentheses.

**Table S7:** The extent of haplotype sharing between Soays and domestic sheep breeds around genome-wide significant SNPs for hindleg length on chromosomes 16 and 19.

|  | **A) Chromosome 16** | |  |  | | |  | |  |  |  |  |  |  |  |  |
| --- | --- | --- | --- | --- | --- | --- | --- | --- | --- | --- | --- | --- | --- | --- | --- | --- |
|  |  | **SNP name and physical position** | | | | | | | | |  |  |  |  |  |  |
|  |  | **s10403.1** | **s20693.1** | | **s22142.1** | **s23172.1** | | **OAR16_75733335.1** | | **s34571.1** |  |  |  |  |  |  |
| **Effect on leg length** | **Haplotype** | **69532333** | **69657258** | | **69679810** | **69726554** | | **69758745** | | **69853299** | **Haplotype count^a^** | **Haplotype frequency^b^** | **Global HS mean (SD)^c^** | **Breed with max HS^d^** | **Max HS^e^** | **Sheep HapMap breeds^f^** |
| Short | 1 | G | G | | A | G | | G | | G | 536 | 0.046 | 0.657(0.505) | BOR | 2.747 | 42 |
| Long | 2 | G | A | | G | A | | G | | A | 6553 | 0.564 | 0.641(0.399) | NTX | 1.252 | 19 |
| Long | 3 | G | A | | G | A | | A | | A | 1553 | 0.134 | 0.612(0.386) | BSI | 1.332 | 50 |
| Long | 4 | A | A | | G | A | | A | | G | 1396 | 0.12 | 0.72(0.406) | NDZ | 1.866 | 46 |
| Long | 5 | G | A | | G | A | | A | | G | 683 | 0.059 | 0.739(0.532) | BOR | 1.41 | 33 |
| Long | 6 | G | G | | G | A | | G | | A | 614 | 0.053 | 0.614(0.455) | NSP | 1.355 | 21 |
| Long | 7 | G | G | | G | A | | G | | G | 228 | 0.02 | 0.755(0.527) | BBB | 1.996 | 5 |
| Long | 8 | A | A | | G | A | | A | | A | 18 | 0.002 | 0.578(0.364) | RMB | 1.244 | 56 |
| Long | 9 | A | A | | G | A | | G | | A | 18 | 0.002 | 0.651(0.373) | CME | 1.178 | 23 |
| Long | 10 | G | A | | G | A | | G | | G | 10 | 0.001 | 0.563(0.148) | NSP | 0.818 | 6 |
|  |  |  |  | |  |  | |  | |  |  |  |  |  |  |  |
|  | **B) Chromosome 19** | |  | |  |  | |  | |  |  |  |  |  |  |  |
|  |  | **SNP names and physical position** | | | | | | | | |  |  |  |  |  |  |
|  |  | **s49822.1** | **s47390.1** | **OAR19_55177848.1** | | | **s74894.1** | | **s72304.1** | **s09369.1** |  |  |  |  |  |  |
|  | **Haplotype** | **52141808** | **52180988** | **52280918** | | | **52470202** | | **52497397** | **52697161** | **Haplotype count** | **Haplotype frequency** | **Global HS mean (SD)** | **Breed with max HS** | **Max HS** | **Sheep HapMap breeds** |
| Short | 1 | G | A | G | | | A | | G | G | 1072 | 0.092 | 1.51(0.494) | BOR | 4.019 | 5 |
| Short | 2 | G | A | G | | | A | | G | A | 70 | 0.006 | 0.933(0.294) | MER | 1.024 | 5 |
| Short | 3 | G | A | A | | | A | | G | G | 34 | 0.003 | 1.191(0.346) | LAC | 1.127 | 13 |
| Long | 4 | G | A | G | | | G | | G | G | 3922 | 0.338 | 0.835(0.36) | BHM | 1.853 | 40 |
| Long | 5 | G | A | A | | | G | | A | G | 2949 | 0.254 | 1.504(1.132) | BOR | 1.932 | 15 |
| Long | 6 | G | A | A | | | G | | G | A | 1383 | 0.119 | 1.09(0.291) | BBB | 1.975 | 63 |
| Long | 7 | A | G | A | | | G | | G | A | 972 | 0.084 | 1.034(0.22) | FIN | 1.592 | 59 |
| Long | 8 | G | A | A | | | G | | G | G | 788 | 0.068 | 1.091(0.475) | SWA | 1.536 | 52 |
| Long | 9 | G | A | A | | | G | | A | A | 322 | 0.028 | 0.858(0.302) | BOR | 2.367 | 35 |
| Long | 10 | G | A | G | | | G | | G | A | 68 | 0.006 | 0.749(0.195) | RMB | 1.472 | 60 |
| Long | 11 | A | G | A | | | G | | G | G | 22 | 0.002 | 0.912(0.32) | COM | 2.034 | 42 |

^a^ Number of haplotypes in the Soay sheep (n= 5,805 )

^b^ Haplotype frequency in the Soay sheep

^c^ Mean and standard deviation (SD) of haplotype sharing (HS) with Soays for each core haplotype in all non-Soay breeds.

^d^ Sheep breed with largest observed mean haplotype sharing with Soay sheep.

^e^ Maximum observed mean haplotype sharing with Soays for each core haplotype

^f^ Number of sheep breeds in the ovine HapMap project that were found to share each core haplotype identified in Soays (out of N= 73).

¶

**Fig S1:** Correlation of variance explained by each chromosome between traits. Values above the diagonal show the Pearson's correlation coefficient and P values for each pairwise combination of traits.


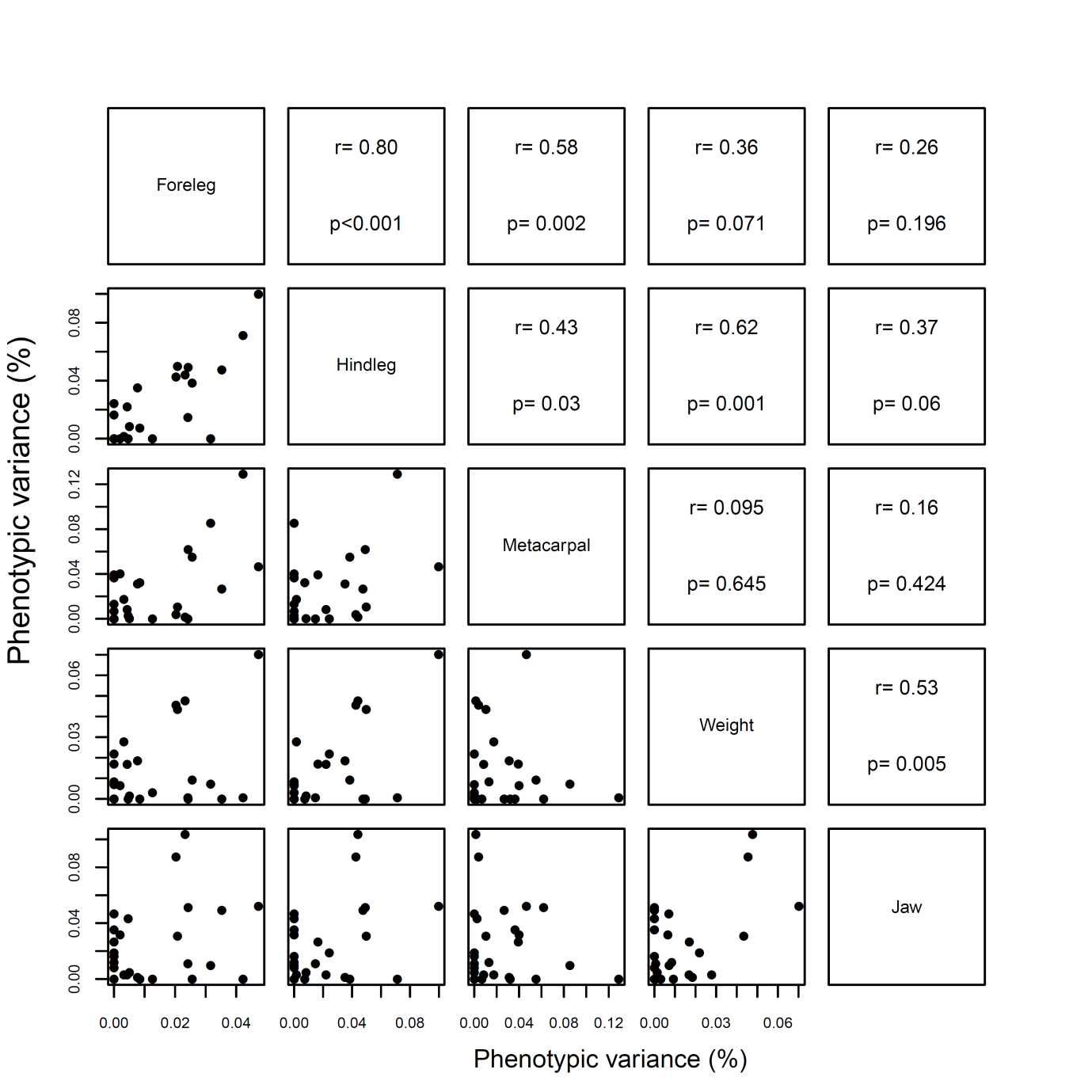


**Fig S2:** Correlation of variance explained by each 150 SNP window between traits. Values above the diagonal show the Pearson's correlation coefficient and P values for each pairwise combination of traits.


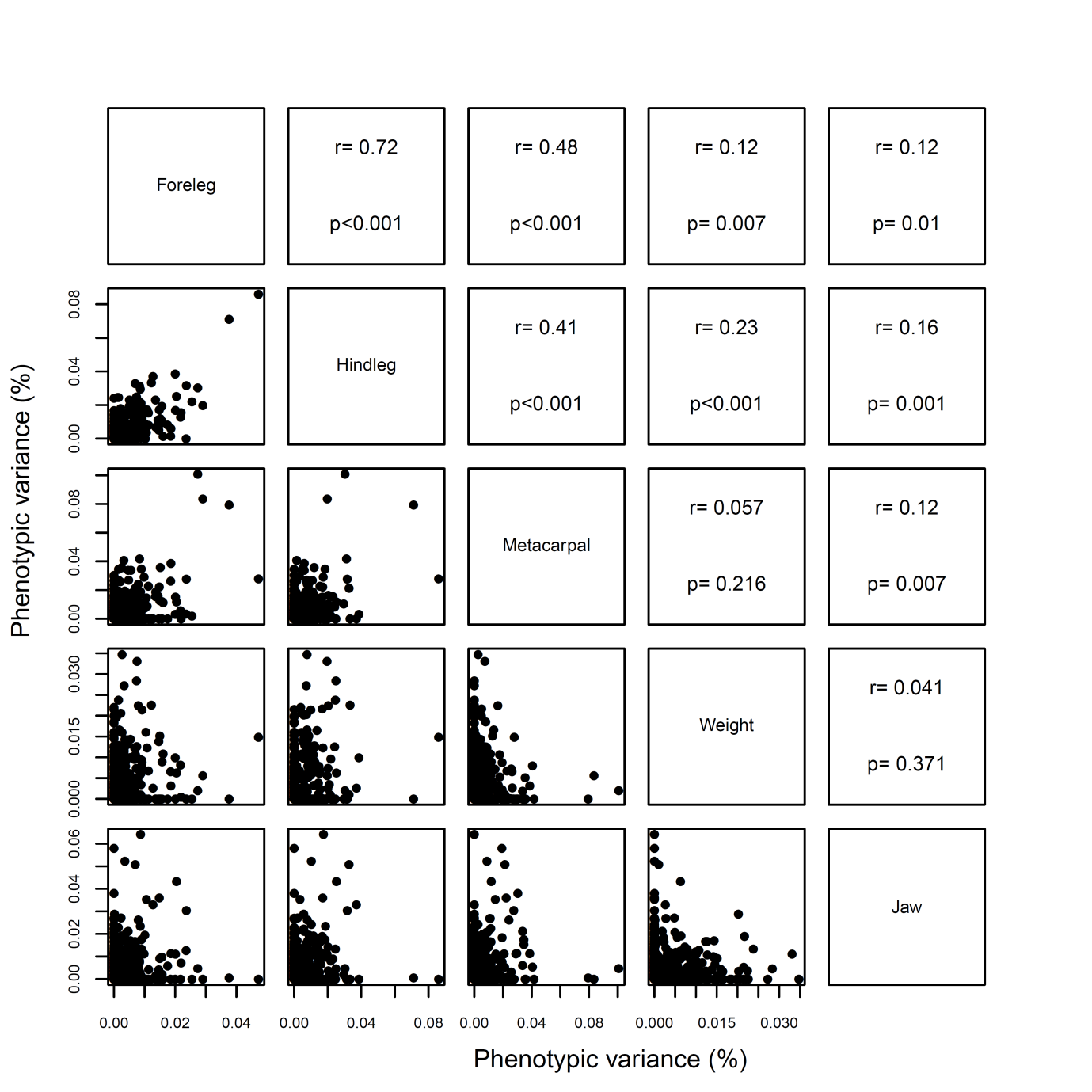


**Fig S3:** Population frequency of A) s23172.1 and C) s74894.1 in Village Bay between 1990 and 2012. Histograms show the distribution of regression slopes from 1000 gene-drop simulations for B) s23172.1 and D) s74894.1. The solid black lines indicate the observed slopes.


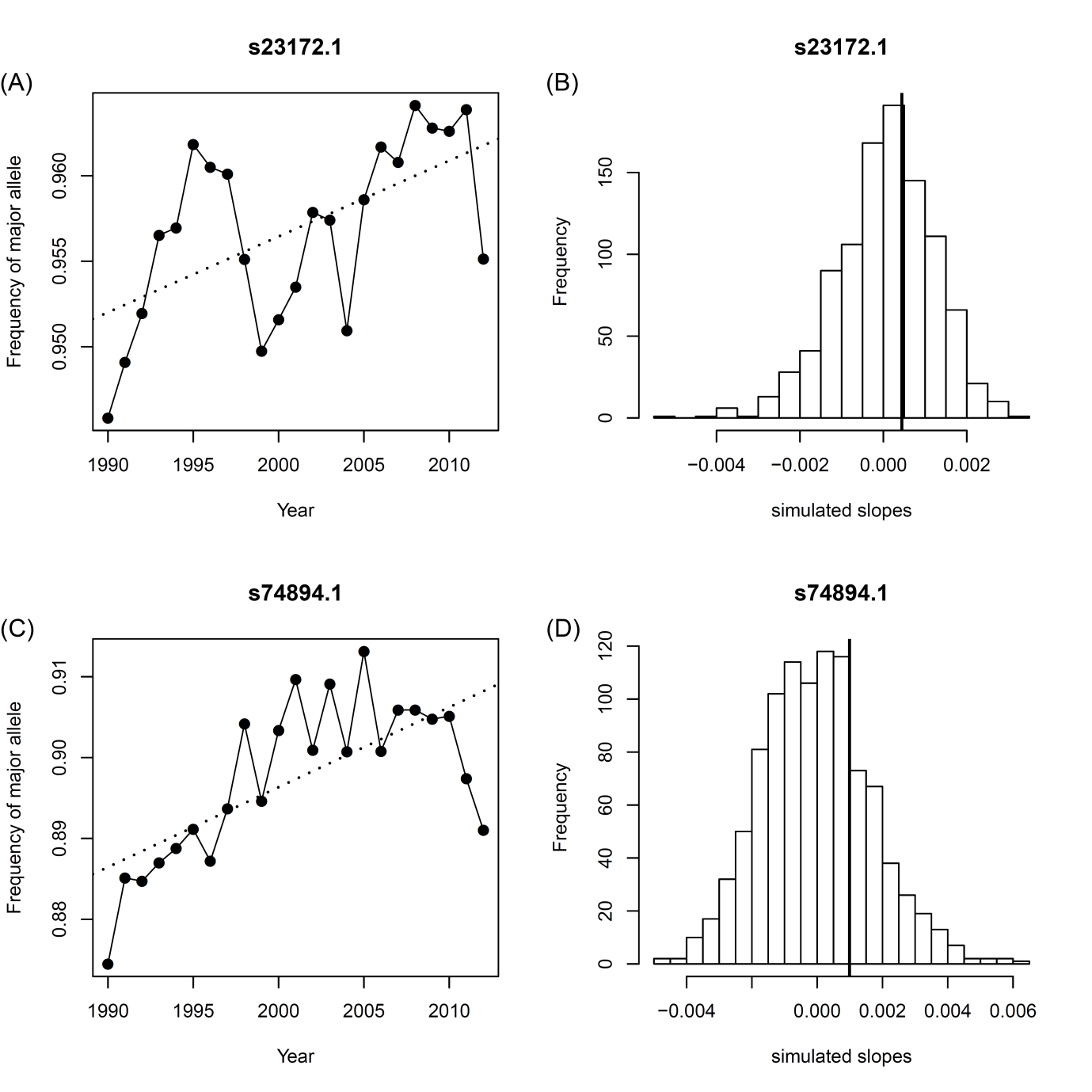


**Fig S4:** Heatmaps of pairwise LD between SNPs within 1 MB either side of the SNPs on chromosome 16 and 19 showing strongest association with metacarpal length. LD was calculated as r^2^. Red dots depict SNPs which were significantly associated with one or more leg length traits. The physical distance between the first and last SNP shown around s23172.1 and s74894.1 was 1.94Mb and 1.87Mb respectively. **
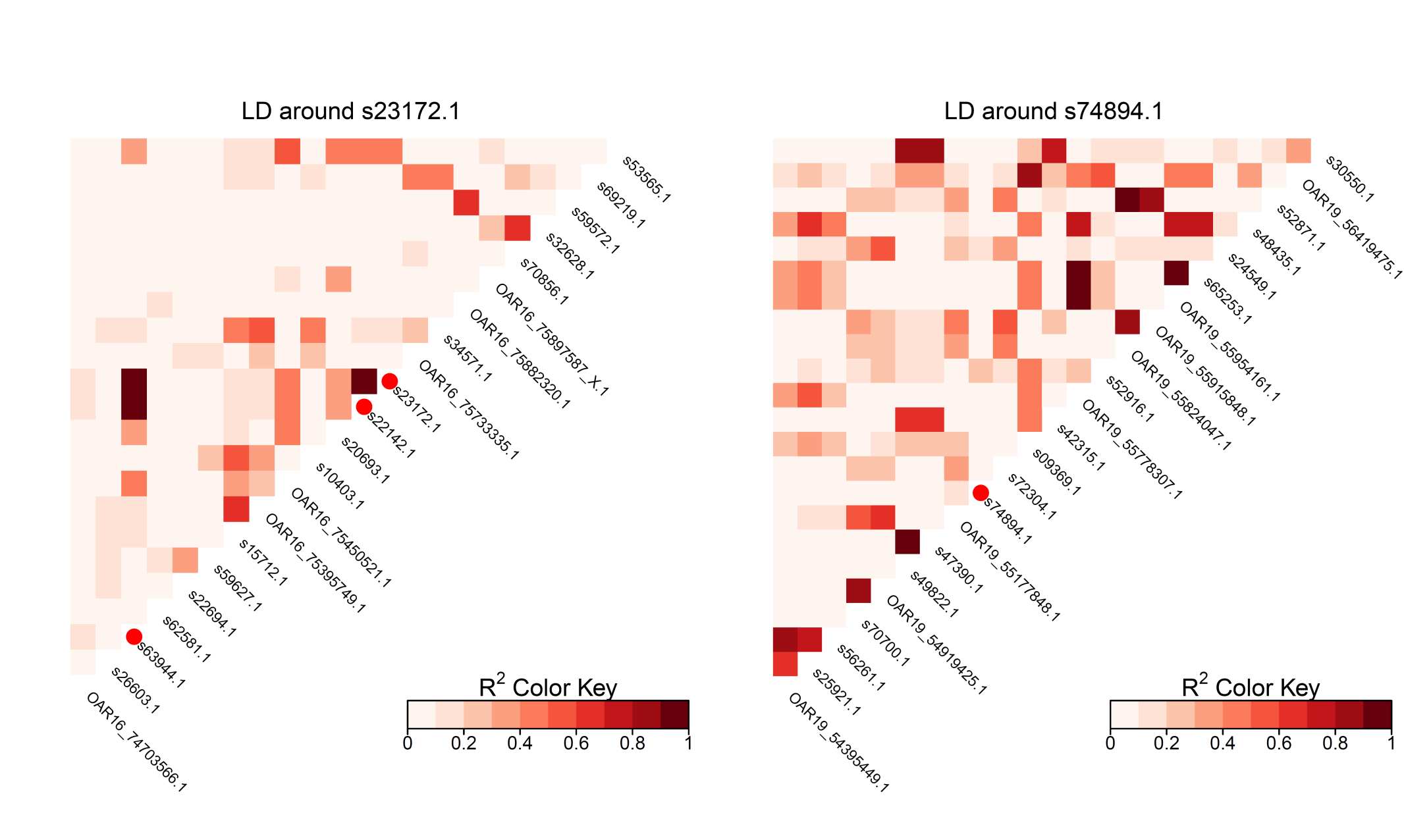
**

**Fig S5:** Unique haplotypes in the Soay sheep population in a region within 1 MB either side of s23172.1 on chromosome 16 and s74894.1 on chromosome 19 showing strongest association with metacarpal length. The physical distance between the first and last SNP shown around s23172.1 and s74894.1 was 1.94Mb and 1.87Mb respectively. Only haplotypes with count >5 are shown. Light grey cells indicate that alleles at that SNP were identical to the allele in the most common unique haplotype, and dark grey cells indicate that alleles at that position differed. Haplotypes which harboured long-leg alleles at the SNP showing strongest associations are shown at the top, haplotypes harbouring short-leg alleles at the bottom (shown with the blue bars on the left of each region), and haplotypes are sorted by frequency. The number of observations are shown for each haplotype.


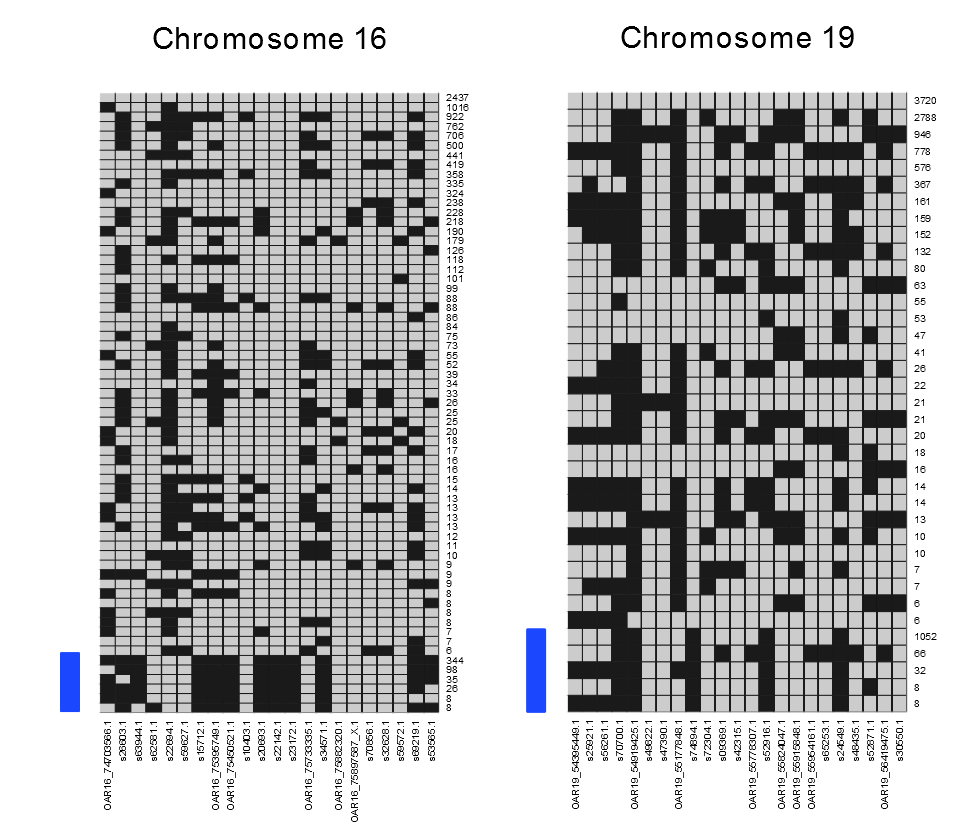


**Literature cited**

Bérénos C, Ellis PA, Pilkington JG, Pemberton JM (2014) Estimating quantitative genetic parameters in wild populations: a comparison of pedigree and genomic approaches. *Molecular Ecology* **23**, 3434-3451.

Gratten J, Pilkington JG, Brown EA*, et al.* (2012) Selection and microevolution of coat pattern are cryptic in a wild population of sheep. *Molecular Ecology* **21**, 2977-2990.
